# Phosphorylation of the Synaptonemal Complex Protein Zip1 Regulates the Crossover/Noncrossover Decision during Yeast Meiosis

**DOI:** 10.1371/journal.pbio.1002329

**Published:** 2015-12-18

**Authors:** Xiangyu Chen, Ray T. Suhandynata, Rima Sandhu, Beth Rockmill, Neeman Mohibullah, Hengyao Niu, Jason Liang, Hsiao-Chi Lo, Danny E. Miller, Huilin Zhou, G. Valentin Börner, Nancy M. Hollingsworth

**Affiliations:** 1 Biochemistry and Cell Biology, Stony Brook University, Stony Brook, New York, United States of America; 2 Center for Gene Regulation in Health and Disease and Department of Biological Sciences, Cleveland State University, Cleveland, Ohio, United States of America; 3 Molecular Biology Program, Howard Hughes Medical Institute, Memorial Sloan-Kettering Cancer Center, New York City, New York, United States of America; 4 Howard Hughes Medical Institute, Memorial Sloan-Kettering Cancer Center, New York City, New York, United States of America; 5 Molecular and Cellular Biochemistry, Indiana University, Bloomington, Indiana, United States of America; 6 Ludwig Institute for Cancer Research, University of California, San Diego, La Jolla, California, United States of America; 7 Chemistry and Biochemistry, University of California, San Diego, La Jolla, California, United States of America; 8 Stowers Institute for Medical Research, Kansas City, Missouri, United States of America; 9 Department of Molecular and Integrative Physiology, University of Kansas Medical Center, Kansas City, Kansas, United States of America; 10 Cellular and Molecular Medicine, University of California, San Diego, La Jolla, California, United States of America; Mount Sinai Hospital, CANADA

## Abstract

Interhomolog crossovers promote proper chromosome segregation during meiosis and are formed by the regulated repair of programmed double-strand breaks. This regulation requires components of the synaptonemal complex (SC), a proteinaceous structure formed between homologous chromosomes. In yeast, SC formation requires the “*ZMM*” genes, which encode a functionally diverse set of proteins, including the transverse filament protein, Zip1. In wild-type meiosis, Zmm proteins promote the biased resolution of recombination intermediates into crossovers that are distributed throughout the genome by interference. In contrast, noncrossovers are formed primarily through synthesis-dependent strand annealing mediated by the Sgs1 helicase. This work identifies a conserved region on the C terminus of Zip1 (called Zip1 4S), whose phosphorylation is required for the *ZMM* pathway of crossover formation. Zip1 4S phosphorylation is promoted both by double-strand breaks (DSBs) and the meiosis-specific kinase, *MEK1/MRE4*, demonstrating a role for *MEK1* in the regulation of interhomolog crossover formation, as well as interhomolog bias. Failure to phosphorylate Zip1 4S results in meiotic prophase arrest, specifically in the absence of *SGS1*. This gain of function meiotic arrest phenotype is suppressed by *spo11Δ*, suggesting that it is due to unrepaired breaks triggering the meiotic recombination checkpoint. Epistasis experiments combining deletions of individual *ZMM* genes with *sgs1-md zip1-4A* indicate that Zip1 4S phosphorylation functions prior to the other *ZMM*s. These results suggest that phosphorylation of Zip1 at DSBs commits those breaks to repair via the *ZMM* pathway and provides a mechanism by which the crossover/noncrossover decision can be dynamically regulated during yeast meiosis.

## Abstract 2

Crossovers between homologous chromosomes are needed for correct segregation during meiosis. This study reveals that Mek1-dependent phosphorylation of the yeast synaptonemal complex protein Zip1 is needed for the decision to resolve recombination intermediates into crossovers rather than noncrossovers.

## Author Summary

During meiosis, double-strand breaks (DSBs) are generated in a programmed fashion to promote recombination between homologs. These programmed DSBs can be repaired either as crossovers or noncrossovers. In normal budding yeast meiosis, most crossovers are formed by a functionally diverse group of “Zmm” proteins that promote pairing of the homologous chromosomes (a process known as “synapsis”), as well as the distribution of crossovers throughout the genome. One member of this group is Zip1, the transverse filament protein of the synaptonemal complex (a meiosis-specific structure formed between the homologous chromosomes). This work shows that phosphorylation of the C terminus of Zip1 is required for synapsis and the formation of interfering crossovers. In the absence of this phosphorylation, noncrossover formation is increased, indicating that Zip1 phosphorylation could provide a mechanism for regulating crossover homeostasis. Genetic experiments indicate that this Zip1 phosphorylation event functions prior to other *ZMM* genes and suggests two functions for Zip1, (1) recruitment to DSBs where phosphorylation promotes formation of synaptic initiation complexes containing other Zmm proteins, and (2) polymerization of Zip1 to form the synaptonemal complex. Phosphorylation is dependent upon the meiosis-specific kinase Mek1, known previously for its role in interhomolog bias. The fact that Mek1 also regulates interfering interhomolog crossover formation raises the possibility that it is the link by which interhomolog bias and crossovers are coordinated during meiosis.

## Introduction

During meiosis, crossovers (COs), in combination with sister chromatid cohesion, physically connect homologous chromosomes, thereby promoting proper segregation at Meiosis I (MI). COs arise by the regulated repair of programmed double-strand breaks (DSBs), formed by the topoisomerase-like protein, Spo11 (SGD S000001014) [1]. This regulation involves components of the synaptonemal complex (SC), a meiosis-specific tripartite structure formed between homologous chromosomes [2].

SC formation begins by condensation of sister chromatids upon protein cores to form axial elements (AEs). AEs contain loops of chromatin tethered at their bases by meiosis-specific axis proteins, which in yeast include Hop1 (SGD S000001334), Red1 (SGD S000004253), and the cohesin kleisin subunit, Rec8 (SGD S000006211) [3–5]. Hotspot sequences are brought to the axes where DSBs result in the recruitment and activation of the meiosis-specific kinase, Mek1 (SGD S000005878), via Mec1/Tel1 (SGD S000000340/ S000000184) phosphorylation of Hop1 [1,6,7]. Mek1 promotes interhomolog (IH) recombination by antagonizing cohesion around DSBs and inhibiting the strand exchange activity of the mitotic recombinase, Rad51 (SGD S000000897), thereby facilitating strand invasion of homologous chromosomes by the meiosis-specific Dmc1 (SGD S000000981) recombinase [8–11]. For many organisms such as yeast and mammals, strand invasion is critical for bringing homologs together. Synapsis is achieved when homologous AEs are held together by the insertion of transverse filament (TF) proteins in the central region to form the SC [2]. In yeast, the TF protein is Zip1 (SGD S000002693) [12].

**Fig 1:**
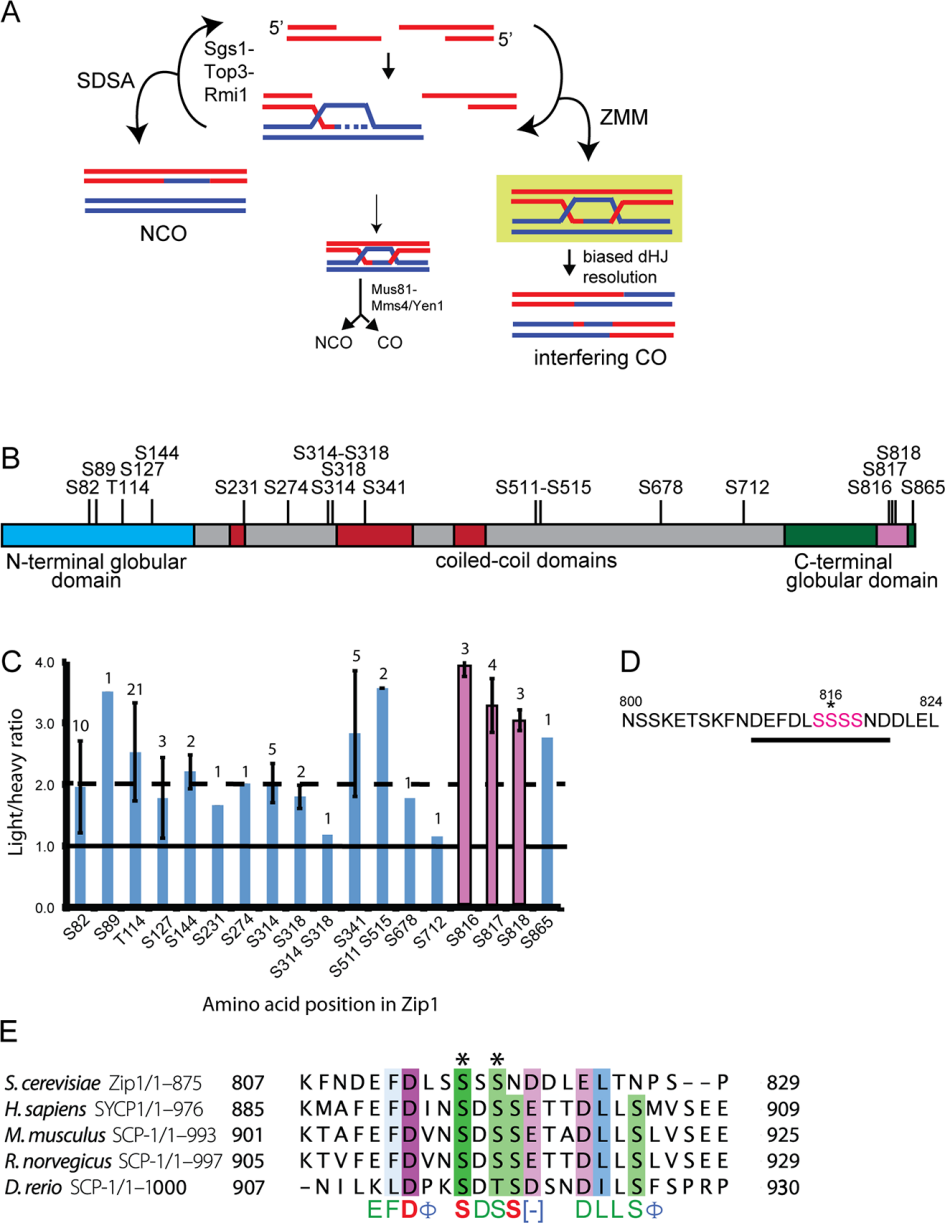
Identification of putative Mek1-regulated phosphosites on Zip1. (A) Different pathways of meiotic DSB repair in yeast (adapted from [18]). Red and blue lines represent duplexes of DNA from the nonsister chromatids of a pair of homologous chromosomes. The green box indicates “stabilization” of IH JMs leading to biased resolution as COs. (B) Schematic showing phosphosites on Zip1 detected from a stable isotope labeling of amino acids in cell culture (SILAC) experiment using the *mek1-as ndt80Δ* diploid, NH2221. A hyphen indicates both amino acids were phosphorylated on the same peptide. Gray boxes indicate predicted coiled-coil domains [12]. The pink box represents the 800–824 amino acid region required for synapsis [12]. (C) Average Light/Heavy (L/H) ratios of Zip1 phosphopeptides detected by SILAC. Ratios above the dashed line indicate possible regulation by Mek1. Error bars indicate the standard deviation for the L/H ratios in cases where more than one phosphopeptide was detected. Numbers above each bar indicate how many peptides containing the indicated phosphosite were observed. Pink indicates phosphosites in the 4S region. (D) Sequence of the region indicated by the pink box in B. Pink color indicates the serines mutated in the *zip1-4A* and *zip1-4D* alleles. Antibodies against phosphorylated S816 (indicated by an asterisk) were generated using the peptide underscored by the black line. (E) Clustal Omega alignment of TF proteins from different organisms in the Zip1 4S region. Numbers indicate amino acid position. Dashes indicate gaps. In the consensus, red = identical amino acids, green = amino acid is present in four species, blue = amino acids with conserved properties, ϕ = hydrophobic, [–] = acidic. Asterisks (*) represent residues predicted by NetPhos analysis to be phosphorylated in all five species.

Synapsis requires the stabilization of strand invasion intermediates by a functionally diverse set of proteins, including Zip1, Zip2 (SGD S000003218), Zip3 (SGD S000004386), Zip4/Spo22 (SGD S000001335), Msh4 (SGD S000001891), Msh5 (SGD S000002313), Mer3 (SGD S000003220), and Spo16 (SGD S000001196), which are encoded collectively by the *ZMM* genes [13] (Fig 1A). The IH connections made by Zmm proteins promote down-regulation of Spo11 activity [14]. DSBs processed along the *ZMM* pathway form double Holliday junctions (dHJs) that are asymmetrically resolved to produce COs [13,15] (Fig 1A). *ZMM*-promoted COs are distributed throughout the genome by a phenomenon called genetic interference [16]. Sgs1 (SGD S000004802), the Bloom syndrome RecQ helicase (BLM) ortholog in yeast, works with Top3 (SGD S000004224) and Rmi1 (SGD S000005945) as a “recombination intermediate chaperone” by promoting the disassembly of strand invasion intermediates [17–21]. In wild-type (WT) meiosis, noncrossovers (NCOs) are formed primarily by synthesis-dependent strand annealing (SDSA), in which the invading strand is extended, displaced by the action of the Sgs1-Top3-Rim1, and annealed to the other side of the break [15,22] (Fig 1A). In the absence of *SGS1*, *TOP3*, or *RMI1*, joint molecules (JMs) are formed that are resolved in an unbiased manner to give both COs and NCOs by structure-specific endonucleases such as Mus81-Mms4 (SGD S000002794-S000000302) and Yen1 (SGD S000000843) [17,18,20,21]. Inactivation of *SGS1* partially restores COs to some *zmm* mutants, supporting the idea that one function of the *ZMM*s is to protect recombination intermediates from Sgs1 [19,23].

In yeast, whether a DSB is repaired as a CO or NCO has been proposed to be determined at, or just after, the time of DSB formation [13,24]. Furthermore, there is plasticity in the decision as evidenced by the existence of CO homeostasis [25]. When DSBs are reduced, CO levels are maintained at the expense of NCOs. This behavior suggests that mechanisms exist for regulating the CO/NCO decision after DSBs have been made, but the molecular bases for such regulation have been elusive.

Phosphorylation is a dynamic modification used to regulate different steps in recombination, in addition to IH bias. For example, the highly conserved cell cycle kinases Cdc28-Clb5/6 (Cdk1) (SGD S000000364-S000006324/S000003341) and Cdc7-Dbf4 (Dbf4-dependent kinase; DDK) (SGD S000002175-S000002459) are required for DSB formation, and dHJ resolution is triggered by the polo-like kinase Cdc5 (SGD S000004603) [26–28]. Whether Mek1 or DDK is directly involved in forming IH COs was unknown, because inactivation of these kinases prevents creation of IH recombination intermediates. In this work, phosphoproteomic analysis reveals conserved phosphorylation sites on the C terminus of Zip1 that are required for the *ZMM* pathway of DSB repair. Phosphorylation of these sites is dependent upon *MEK1*, revealing a previously unknown role for this kinase in the regulation of IH recombination and suggesting a link by which IH bias and the CO/NCO decision can be coordinated.

## Results

### Zip1 Is Phosphorylated on Its C Terminus in a Region Required for Chromosome Synapsis

To identify phosphorylation sites regulated by Mek1, stable isotope labeling of amino acids in cell culture (SILAC) was performed using a *mek1-as ndt80Δ* diploid as described in [29]. *mek1-as* encodes a conditional version of Mek1 that can be inactivated by the purine analog 1-NA-PP1 [30]. Diploids lacking the meiosis-specific transcription factor *NDT80* (SGD S000001166) arrest in meiotic prophase with fully synapsed chromosomes and unresolved dHJs [15,31]. A *mek1-as ndt80Δ arg4 lys4* diploid was grown in synthetic medium containing “heavy” or “light” versions of arginine and lysine and then transferred to Spo medium for 14 h. Mek1-as was inhibited in the heavy culture by addition of 1-NA-PP1 for 20 min, chromatin was isolated from both cultures, and equal amounts of protein were mixed and digested with trypsin. Phosphopeptides were enriched using immobilized metal affinity chromatography and analyzed by mass spectrometry (MS). Non-Mek1-dependent phosphopeptides should be present in equal amounts in both cultures giving light:heavy (L/H) peptide ratios of 1. Mek1-dependent phosphopeptides are predicted to be under-represented in the heavy culture and exhibit L/H ratios > 1. This approach revealed that one phosphoprotein potentially regulated by Mek1 is the TF protein Zip1.

MS analysis identified 18 phosphorylation sites on Zip1 (Fig 1B). Two of these phosphopeptides (S314 S318 and S712) exhibited L/H ratios around 1.0, indicating that equivalent amounts of Zip1 protein were present in the light and heavy cultures (Fig 1C). Of particular interest were three out of four adjacent serine residues in the C terminus (S815-S818, hereafter referred to as 4S) that exhibited L/H ratios >3, suggesting potential regulation by Mek1. These serines are located within a 25-amino acid region required for chromosome synapsis and WT levels of recombination, suggesting that phosphorylation of Zip1 at the C terminus plays a role in these processes (Fig 1B and 1D) [12].

Although TF proteins are structurally similar, they are poorly conserved at the primary sequence level [2]. It is therefore compelling that the 4S region of Zip1 is conserved among yeast, mammals and zebrafish TF proteins (Fig 1E). Furthermore, NetPhos analysis [32] predicts that at least two of the residues in the conserved 4S region are phosphorylated in all five species. In contrast, no homology was observed in this region for the TF proteins from *Drosophila melanogaster*, *Caenorhabditis elegans*, or *Arabidopsis thaliana*.

### Phosphorylation of Zip1 4S Promotes Negative Feedback Regulation of Spo11

The functional significance of Zip1 4S phosphorylation was tested using *ZIP1* alleles in which all four serines were mutated either to alanine (*zip1-4A*) or aspartic acid (*zip1-4D*) to prevent or mimic phosphorylation, respectively. The *zip1-4A* diploid exhibited spore viability that is significantly reduced compared to WT (76.7% vs. 94.3%), but which is higher than the *zip1Δ* (45.9%) (*p* < 0.0001) (S1 Table). This decrease in spore viability is due the absence of negative charges as the *zip1-4D* diploid produced 96.2% viable spores. Meiotic time course analysis of steady state Zip1 protein levels revealed that 4S phosphorylation is required for Zip1 degradation (S1A Fig). The increased stability of Zip1-4A protein raised the possibility that the spore viability defect was indirectly due to the persistence of Zip1-4A. If this were true, then the *zip1-4A* mutant phenotype should be dominant, since the Zip1-4A protein should fail to get degraded even in the presence of the WT protein. In fact, the amount of Zip1 protein persists at higher levels in the *ZIP1/zip1-4A* heterozygote than the WT strain, yet spore viability in the heterozygote is indistinguishable from WT (94.5% *ZIP1/zip1-4A* vs 94.3% *ZIP1*; *p* = 0.149) (S1A Fig, S1 Table). Therefore, the failure to degrade Zip1-4A is not the reason for its mutant phenotype.

**Fig 2:**
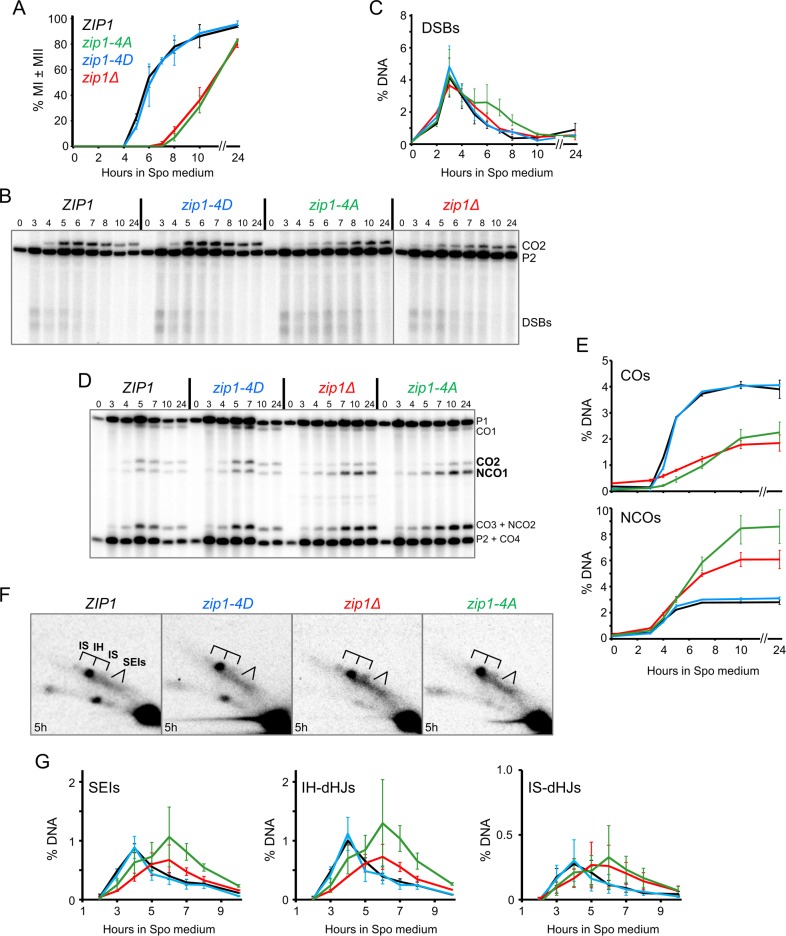
Meiotic time course analysis of various *zip1* diploids. Homozygous WT (NH2297::p382^2^), *zip1-4D* (NH2297::p382-4D^2^), *zip1Δ* (NH2297::pRS304^2^), and *zip1-4A* (NH2297::p382-4A^2^) diploids were sporulated and cells taken at different time points. (A) Meiotic progression was measured by counting the percentage of binucleate (MI) and tetranucleate (MII) cells after staining with 4’, 6-diamidino-2-phenylindole (DAPI). (B) DSBs detected by XhoI digestion of genomic DNA was probed with a fragment to detect the *HIS4*::*LEU2* hotspot. (C) Quantification of DSBs from two different time courses, one of which is shown in Panel B. (D) COs and NCOs detected by XhoI/NgoMIV digestion of the DNA. (E) Quantification of CO2 and NCO1 as in Panel C. (F) Representative examples of JMs at the indicated time points. (G) Quantification of single end invasions (SEIs), IH, and intersister (IS) dHJs as in Panel C. Complete gels are shown in S2 Fig. For all panels, average values from two independent time courses are shown. Error bars indicate the range.

Meiotic progression was delayed in *zip1-4A* and *zip1Δ* diploids, but not *zip1-4D*, providing further evidence that negative charges at these positions are important for *ZIP1* function (Fig 2A). Physical analysis using the *HIS4*::*LEU2* hotspot on chromosome III was used to monitor DSB formation and repair [24]. This hotspot consists of a DSB site flanked by polymorphic XhoI restriction sites (S2A Fig). DSBs can be distinguished by Southern blot analysis using XhoI-digested DNA. DSBs in *zip1-4A* appeared at the same time as WT and *zip1-4D*, but were present in greater abundance than the *zip1Δ* at later time points and took longer to disappear (Fig 2B and 2C).

**Fig 3:**
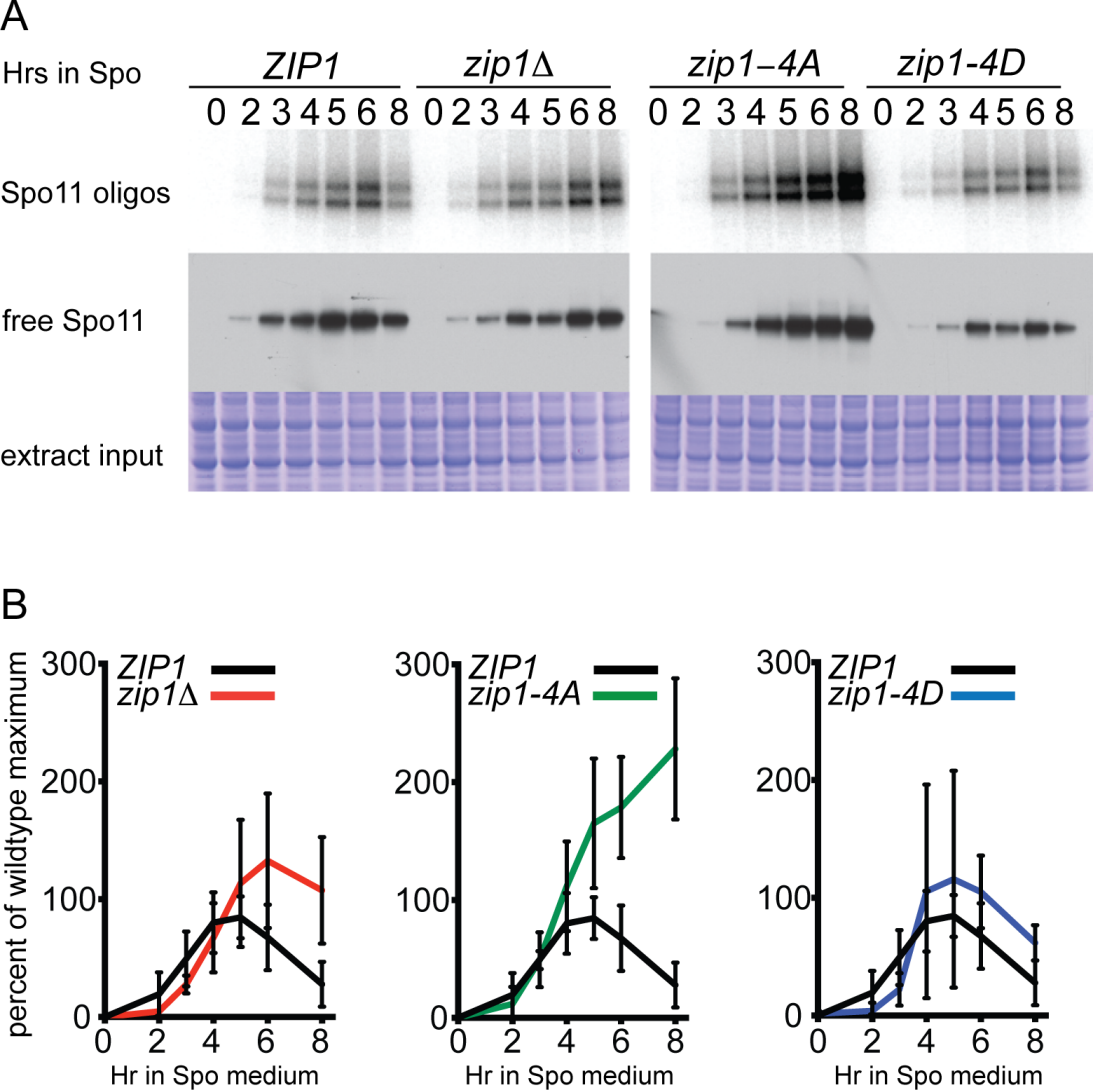
Quantification of DSBs in various *zip1* mutants using Spo11 oligonucleotides. Time courses of meiotic cultures of *ZIP1* (NH2311), *zip1*::*LEU2* NH2312, *zip1-4A* (NH2313), and *zip1-4D* (NH2314) containing Protein A-tagged Spo11 were performed in parallel as described in [14]. (A) Spo11-Protein A was immunoprecipitated using Protein A antibodies, and the covalently attached radioactively end-labeled oligonucleotides were detected by autoradiography (top panel). Free Spo11 was detected by probing immunoblots of the extracts with Protein A antibodies (middle panel). Extracts used for Spo11 immunoprecipitation were fractionated by SDS-PAGE and proteins stained with Coomassie Brilliant Blue to show that equivalent amounts of extract were used as input for the immunoprecipitation reactions (bottom panel). (B) Quantification of mean Spo11 oligonucleotide levels from four independent experiments. Error bars represent the standard deviation. Mutants are plotted with WT data collected in parallel.

As an alternative way of assessing DSB levels, accumulation of genome-wide Spo11 oligonucleotides was assayed. After DNA cleavage, Spo11 is removed by endonucleolytic digestion, resulting in oligonucleotide “tags” [33]. The *zip1-4A* mutant reproducibly accumulated significantly higher levels of Spo11 oligonucleotides than any of the other strains, including the *zip1Δ* (Fig 3). These results indicate that Spo11 down-regulation due to a failure of *ZMM*-mediated IH engagement is more impaired in the presence of the Zip1-4A protein than in the absence of Zip1 altogether.

### Zip1-4S Phosphorylation Promotes COs and Suppresses NCOs at the *HIS4-LEU2* Hotspot

Double digestion with XhoI and NgoMIV allows detection of both CO and NCO chromosomes at the *HIS4*::*LEU2* hotspot [34] (S2A Fig). COs were reduced to a similar extent in *zip1-4A* and *zip1Δ*. In contrast, NCOs were increased, with an even higher level of NCOs in *zip1-4A* compared to *zip1Δ* (Fig 2D and 2E). Joint molecules (JMs) indicative of single end invasion (SEI) and dHJ intermediates can be detected using two-dimensional gel electrophoresis [24]. Previous work has indicated that *ZIP1*, as well as the other *ZMM* genes, promotes the transition from DSBs into SEI intermediates as well as dHJs [13]. Like *zip1Δ*, SEIs and dHJs were delayed in *zip1-4A* (Fig 2F and 2G) (S2D Fig). The areas under the SEI and IH-dHJ curves appear to be greater in the *zip1-4A* diploid compared to WT and *zip1Δ*, as expected given the increased number of DSBs. It is also possible that intermediates are accumulating due to a slower rate of repair. The formation of intersister (IS) JMs is similarly delayed in both *zip1-4A* and *zip1Δ*. We conclude that phosphorylation of Zip1 4S is responsible for the requirement of *ZIP1* in promoting stable strand invasion intermediates.

### Phosphorylation of Zip1 4S Is Required for Making COs That Are Distributed by Interference

**Fig 4:**
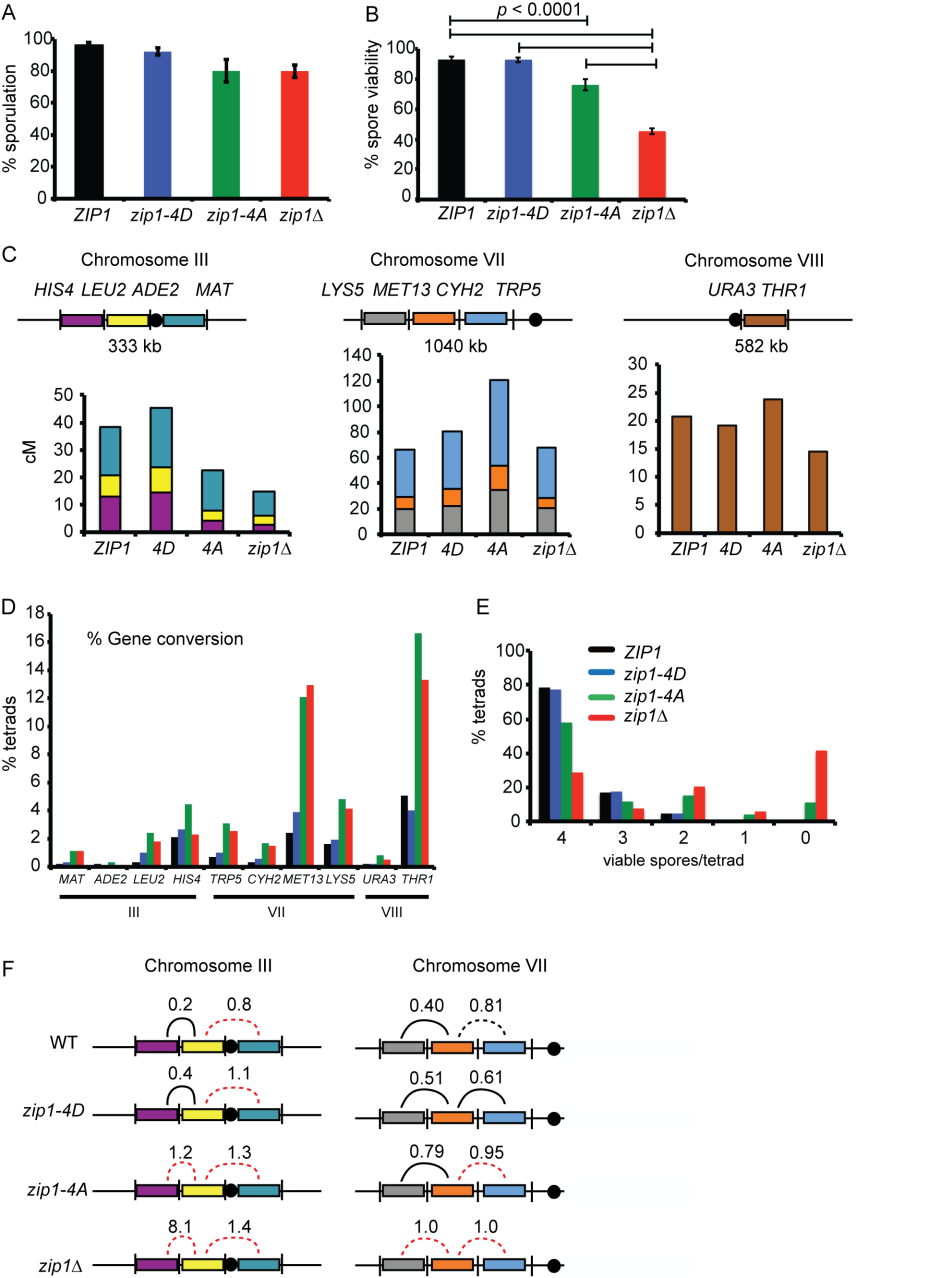
Genetic analysis of sporulation, spore viability, and recombination in various *zip1* diploids. (A) *ZIP1* (NHY957), *zip1-4D* (NH2241::p382), *zip1Δ* (NH2241::pRS304), and *zip1-4A* (NH2241::p382-4A) were sporulated for 2 d at 30°C. Average sporulation from five independent colonies was graphed; error bars indicate the standard deviation. (B) Average spore viability calculated from dissection of nine independent transformants from each diploid. Brackets indicate strain pairs whose spore viability is significantly different (χ^2^; *p* < 0.0001). The number of tetrads dissected for each strain: *ZIP1*, 449; *zip1-4D*, 1,332; *zip1-4A*, 1,857; and *zip1Δ*, 4,012. The isogenic *ZIP1* data shown in Panels B and E were previously published [35]. (C) Map distances for the indicated intervals on Chromosomes III, VII, and VIII were calculated using the formula from Perkins [36]. At least 940 tetrads were analyzed for each genotype. *ZIP1* map distances were generated by pooling data from published NHY957 dissections [25,35,37]. *p*-Values indicating the significance between the map distances of different intervals for mutants and WT are shown in S1 Data. (D) The percent of four viable spore tetrads exhibiting non-2^+^:2^−^ segregation (gene conversion) for the indicated loci. *ZIP1* data were taken from [37]. (E) The distribution of viable spores in tetrads from the dissections shown in Panel B. (F) Interference values for adjacent intervals were calculated using the method of [38]. A solid line indicates interference was observed when either interval was used as the reference. A black broken line indicates interference was detected using one of the reference intervals. A red broken line indicates that no interference was observed using either reference interval. Numbers represent the average of the two ratios for that interval. Data for the *ZIP1* strain in Panel F are from [37]. The aberrantly high value observed for the *zip1Δ* in the *HIS4-LEU2/LEU2-CEN* interval is likely due to the chance occurrence of an extra nonparental ditype (NPD).

To complement the physical analysis, recombination was monitored genetically in a strain containing linked markers on chromosomes III, VII, and VIII [35]. In this strain, sporulation was slightly decreased by *zip1-4A* and *zip1Δ* compared to *ZIP1* and *zip1-4D* (Fig 4A). Consistent with previous results, *zip1-4D* spore viability was equal to WT, while the spore viability of *zip1-4A* was intermediate between WT and the *zip1Δ* (Fig 4B).

Map distances were reduced in the *zip1-4A* mutant on chromosome III, consistent with reduced crossing over measured at the *HIS4*::*LEU2* hotspot (Fig 4C). In contrast, map distances in *zip1-4A* were greater in three intervals on chromosome VII and one interval on chromosome VIII than either WT or *zip1Δ*. Notably, no CO defect was observed for *zip1Δ* on chromosome VII either. This differential effect on CO formation as a function of chromosome size was also observed with a mutant allele of the *ZMM* gene, *ZIP3* [39]. Large chromosomes, such as chromosome VII, sustain disproportionately higher numbers of DSBs in *zmm* mutants [14]. Therefore, the *zip1-4A* increase in COs on chromosomes VII and VIII could be a consequence of continued Spo11 activity generating new breaks, resulting in more CO opportunities. Increasing the number of DSBs should also increase the number of opportunities for gene conversion and in fact, gene conversion events are elevated for loci on all chromosomes in *zip1-4A* (Fig 4D).

A reduction in COs may lead to increased MI non-disjunction due to a failure to physically connect homologs. For both *zip1Δ* and *zip1-4A*, the distribution of viable spores in tetrads supports the idea that spore lethality is due to MI non-disjunction. Both mutants exhibited an increase in the number of 2 viable:2 inviable spores (predicted if one or more pair of homologs have non-disjoined to the same pole) and 0 viable:4 inviable spores (predicted if two or more pairs of homologs have disjoined to opposite poles) (Fig 4E) [40].

Non-disjunction can be directly demonstrated for chromosome III where the codominant *MAT* **a** (SGD S000124955) and *MAT*α (SGD S000029699) alleles produce a distinctive nonmating (NM) phenotype when chromosome III is disomic. Chromosome III non-disjunction (in the absence of recombination) should therefore lead to tetrads with two viable spores, both of which are NMs. Out of 63 two viable spore tetrads obtained from *zip1-4D*, only one contained two NM spores (1.6%, Table 1). In contrast, the number of tetrads exhibiting chromosome III non-disjunction increased to 18.6% in *zip1Δ* and 14.9% in *zip1-4A* (Table 1). Another prediction of MI non-disjunction is that viable spores should be derived from segregation of sister chromatids for the other chromosomes. For chromosome VIII, sister versus nonsister spores can be determined using the centromere-linked marker *URA3* (SGD S000000747) (Table 1). For the *zip1Δ* and *zip1-4A* chromosome III disomic tetrads, equal numbers of either Ura^+^:Ura^+^ or Ura^−^:Ura^−^ sister spores were observed, as expected given that chromosomes III and VIII independently assort (Table 1).

**Table 1:** Chromosome III segregation in two viable spore tetrads from various *zip1* mutants.

Chr. III: *his4 leu2 cen*::*ADE2 MAT*a							Chr. VIII: *cen*::*URA3*				
*HIS4 LEU2 cen MAT* *α*							*cen*				
III	Non-recombinant disomes^b^			Recombinant disomes^c^			% III disomes	III haploid^f^ nonsister spores ADE^+^:Ade^−^	III haploid^f^ nonsister spores Ade^+^:Ade^+^ orAde^−^:Ade^−^	III haploid nonsister/sister^f^	total two viable spore tetrads
VIII*(URA3)*	+:+	−:−	+:−	+:+	−:−	+:−					
*zip1-4D* ^a^	1	0	0	0	0	0	1.6	35	27	1.3	63
*zip1Δ*	70	70	1	1	0	0	18.6^d^	103	520	0.2	765
*zip1-4A*	21	20	0	1	0	0	14.9^e^	83	156	0.5	281

^a^Strains used in these experiments were NH2241::p382-4D, NH2241::pRS304, and NH2241::p382-4A.

^b^Determined by both spores being non-maters and prototrophic for adenine, leucine and histidine.

^c^Determined by both spores being non-maters but auxotrophic for at least one marker in at least one of the spores.

^d^Significantly different from *zip1-4D* (*p* = 0.0007, χ^2^ test).

^e^Significantly different from *zip1-4D* (*p* = 0.007, χ^2^ test).

^f^Determined using two viable spore tetrads in which both spores were haploid (i.e., able to mate).

Recombination was monitored in the intervals between *MAT* and *HIS4* on the disomic III chromosomes. For both *zip1Δ* and *zip1-4A*, all but one of the disomic spores contained III homologs that were non-recombinant between *MAT* and *HIS4* (Table 1). Therefore, failure to phosphorylate Zip1 4S results in reduced COs and increased non-disjunction of chromosome III, similar to *zip1Δ*.

To assess whether non-disjunction is occurring on other chromosomes, the segregation of chromosome III was followed using *ADE2* (SGD S000005654), which was integrated adjacent to the centromere on chromosome III (Table 1). In the two viable spore tetrads that were not disomic for chromosome III (i.e., both spores were maters), random spore death should result in a 2:1 ratio of nonsister spores (Ade^+^: Ade^−^) to sister spores (Ade^+^: Ade^+^ or Ade^−^: Ade^−^) for chromosome III. If, however, spore death is due to non-disjunction of a chromosome(s) other than III, then tetrads containing chromosome III sister spores should be over-represented in the two viable spore asci. The *zip1-4D* diploid exhibited a ratio of 1.3 nonsister/sister spore tetrads, which is not significantly different from the ratio of 2 predicted for random spore death (χ^2^, *p* = 0.357). In contrast, the nonsister/sister spore ratios for *zip1Δ* and *zip1-4A* were 0.2 and 0.5, respectively (Table 1), supporting the idea that non-disjunction of chromosomes other than III also contributes to the spore lethality.

COs from the *ZMM* pathway exhibit genetic interference, where a CO in one interval decreases the likelihood of COs in adjacent intervals [16]. One way of measuring interference is to divide tetrads into two classes based on crossing over in a reference interval: (1) those with no detectable CO in the interval (parental ditypes [PDs]) and (2) those with at least one detectable CO (nonparental ditypes [NPDs] and tetratypes [TTs]) [38]. Map distances for an adjacent test interval are then calculated for each class. If interference is acting, then the Class 2/Class 1 map distance ratio should be <1. In the absence of interference, the ratio should be = 1. With one exception, *zip1-4D* exhibited similar levels of interference as WT for intervals on chromosome III and VII (Fig 4F). In contrast, interference was absent or weakened in the *zip1Δ* and *zip1-4A* diploids on both chromosome III and VII. These data indicate that the bulk of the COs observed in *zip1-4A* occur via the noninterfering CO pathway.

*MUS81* encodes a subunit of a structure-specific endonuclease, which in combination with *MMS4*, is responsible for generating COs along the noninterfering pathway [17,35]. A *mus81Δ* diploid produced 38.7% viable spores, similar to *mus81Δ zip1-4D* (40.0%), while *zip1Δ* exhibited 48.4% (S1 Table). The number of viable spores generated by *mus81Δ zip1Δ* (16.1%) is not significantly different from the number expected for compound defects in two independent CO pathways (17.4%, *p* = 0.35, χ^2^ test). The residual spore viability most likely arises from COs created by alternative structure-specific endonucleases such as Yen1 [17,21]. In contrast, the *mus81 zip1-4A* diploid exhibited 20.2% viable spores, which is significantly lower than the 29.5% predicted based on the 75% spore viability observed for *zip1-4A* (*p* = 0.004, χ^2^ test). The observation that the *mus81Δ zip1-4A* spore viability is similar to that of *mus81Δ zip1Δ* suggests that the higher spore viability in *zip1-4A* is due to *MUS81*-dependent COs.

Zip1 4S phosphorylation promotes interference on both chromosome III and chromosome VII, yet a CO deficit is only observed on the smaller chromosome. This apparent paradox can be explained if Zip1 4S phosphorylation is required for *ZMM*-mediated COs on all chromosomes, but the loss of these COs is obscured on larger chromosomes by an increased number of DSBs allowing the formation of more COs along the alternative *MUS81*-dependent pathway.

### Phosphorylation of Zip1 4S Is Required for Efficient Chromosome Synapsis

**Fig 5:**
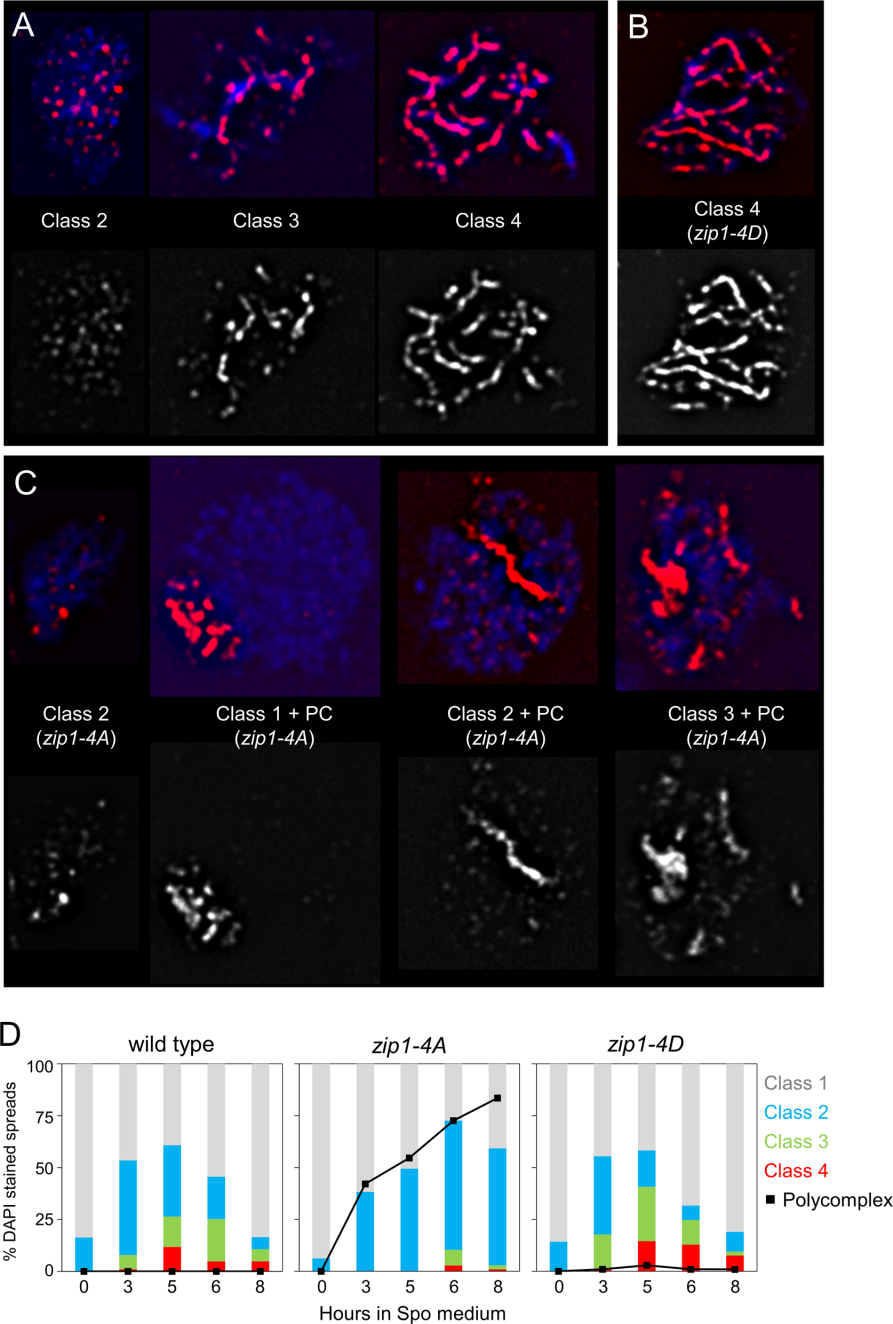
SC formation in various *zip1* mutants. Homozygous WT (NH2297::p382^2^), *zip1-4D* (NH2297::p382-4D^2^), and *zip1-4A* (NH2297::p382-4A^2^) diploids were sporulated and cells taken at different time points and stained with DAPI to detect the chromatin and anti-Zip1 antibodies. Spreads were separated into four classes: 1) no Zip1 staining, (2) Zip1 foci only, (3) Zip1 foci and some lines and (4) fragmented/continuous smooth lines. (A) Spreads from the *ZIP1* diploid showing the different classes of Zip1 staining. (B) A representative Class 4 nucleus from *zip1-4D*. (C) Examples of *zip1-4A* nuclei with PCs as indicated. Upper panels in A, B and C are images in which the DAPI and Zip1 signals were merged. The bottom panels show Zip1 staining alone. (D) Quantification of the different classes observed in meiotic time courses of the WT, *zip1-4D* and *zip1-4A* diploids. At least 100 nuclei were scored for each time point. The black line marks the total percentage of PCs at each time point.

The *ZMM* genes are required for chromosome synapsis [13]. *ZIP1* and *zip1-4D* exhibited similar frequencies and timing of synapsis when monitored by staining spread chromosomes with antibodies against either Zip1 in the SK1 background (Fig 5A, 5B and 5D) or the AE protein, Red1, and the central element protein, Ecm11-myc in the BR background [5,41] (S3 Fig). In contrast, SC formation was delayed and greatly reduced in *zip1-4A*, with the majority of cells exhibiting only Zip1 foci (Fig 5C and 5D; S3 Fig). Defects in synapsis often lead to the formation of polycomplexes (PCs), which contain aggregates of Zip1 not associated with DNA [42]. PCs were abundant specifically in the *zip1-4A* mutant, consistent with a defect in SC formation (Fig 5C and 5D). The presence of Zip1-4A aggregates in PCs may make the protein less accessible to proteases, which could explain the stability of the Zip1-4A protein compared to the WT and Zip1-4D proteins.

### Phosphorylation of Zip1 S816 Is Promoted by DSB Formation

**Fig 6:**
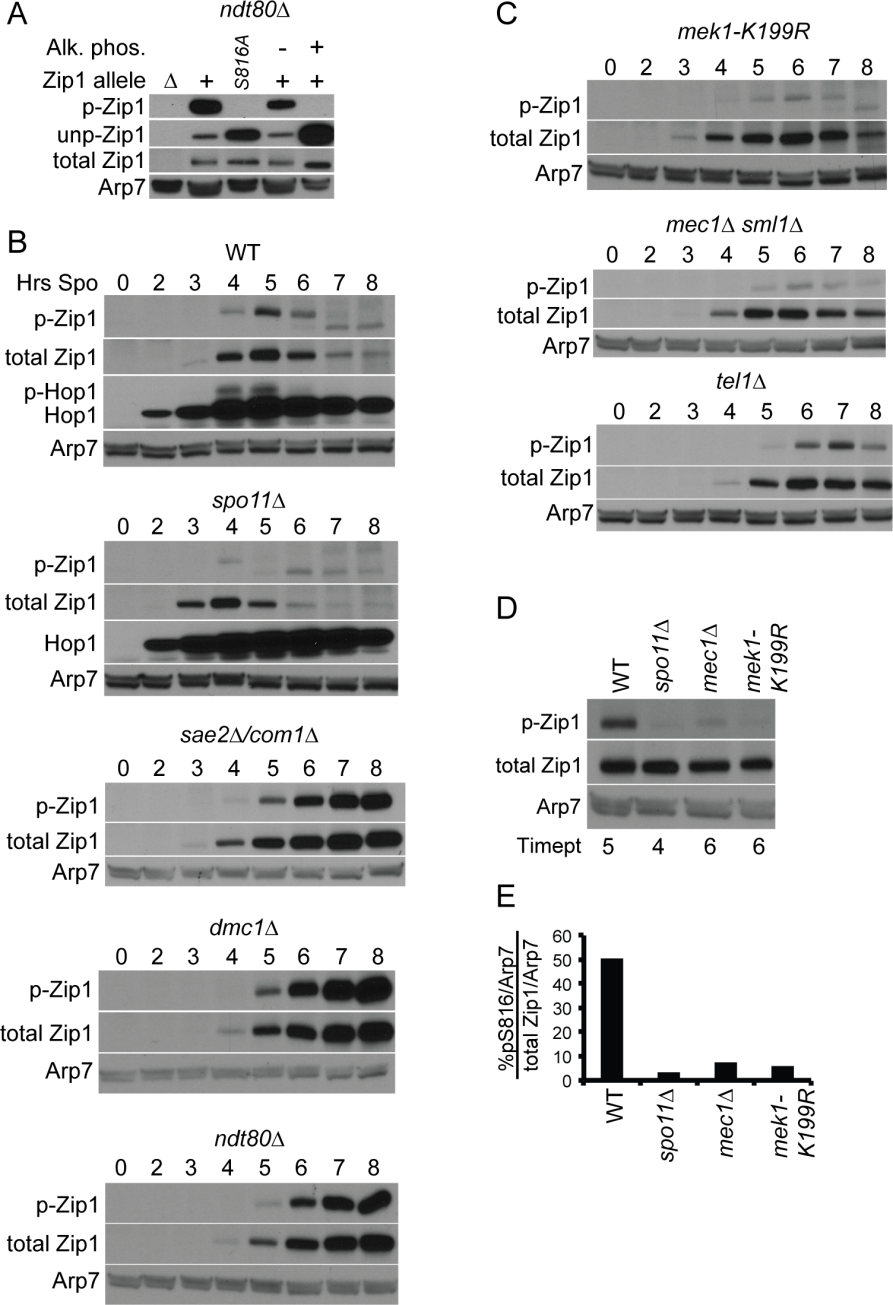
Phosphorylation of Zip1 S816 monitored under different genetic conditions. (A) Protein extracts were generated from *ndt80Δ* (NH2234::p382), *ndt80Δ zip1Δ* (NH2234::pRS304), and *ndt80Δ zip1-S816A* (NH2234::p382-S816A) after 8 h in Spo medium and probed with antibodies specific for Zip1 p-S816 (indicated as p-Zip1) or unphosphorylated Zip1 (indicated as unp-Zip1). In addition, total Zip1 was detected using a polyclonal antibody that recognizes both forms of the proteins. Phosphatase experiments used alkaline phosphatase (AP). Antibodies against Arp7 (SGD S000006238) were used throughout as a loading control [27]. (B) Proteins from mutants defective in different steps of recombination were probed with antibodies that recognize either Zip1 pS816 or total Zip1 protein. For the WT (NH716) and *spo11Δ* (NH1055) strains, Hop1 phosphorylation was used to indirectly assay DSB formation [43]. (*sae2Δ /com1Δ* = NH1054; *dmc1Δ* = NH792; *ndt80Δ* = NH2188) (C) Extracts from *mek1-K199R* (NH566::pLT11::pXC6), *mec1Δ sml1Δ* (SGD S000004523) (NH774), and *tel1Δ* (NH772) diploids were monitored for the presence of Zip1 p-S816 and total Zip1 as in Panel B. (D) Time points with the highest amount of total Zip1 from the indicated mutant time courses shown in Panels B and C were run on the same gel and assayed for the presence of Zip1 p-S816 or total Zip1 to allow for direct comparison. (E) Quantification of the fraction of Zip1 phosphorylated on S816 in Panel D. Total Zip1 and pS816 signals were normalized to the Arp7 signal in the same lane using scanned images of the gel with the ImageQuant 7.0 software and the percent of phosphorylated Zip1 was calculated as pS816/Arp7^pS816^/Total Zip1/Arp7^Total Zip1^ *100.

Antibodies were generated using a peptide containing phosphorylated Zip1 S816 (Fig 1D). Extracts were generated from *ndt80Δ*, *ndt80Δ zip1Δ*, and *ndt80Δ zip1-S816A* strains 8 h after transfer to Spo medium. The *NDT80* gene was deleted to prevent Zip1 degradation [27]. Antibodies specific to phosphorylated S816 (p-S816) exhibited a signal with extracts from *ZIP1* but not *ZIP1-S816A*, and this signal was lost upon phosphatase treatment of the WT protein (Fig 6A). In the course of purifying the phosphospecific antibodies, antibodies that specifically recognize the unphosphorylated form of Zip1 (unp-Zip1) were also obtained. As expected, Zip1 detected by these antibodies displayed a complementary signal to the p-S816 antibodies (e.g., low in the absence of phosphatase and increased after treatment with phosphatase) (Fig 6A). Equivalent amounts of Zip1 protein were present in the different samples, as shown by probing with an antibody that recognizes total Zip1. The Zip1 p-S816 antibodies are therefore highly specific for the phosphorylated protein and provide an excellent tool for monitoring the phosphorylation state of Zip1 at S816.

In WT cells, Zip1 S816 phosphorylation was coincident with DSB formation, indicated indirectly by phosphorylation of Hop1 (Fig 6B) [43]. Zip1 protein was degraded at later time points, as noted previously by [27]. Although a low level of phosphorylation was observed in *spo11Δ*, this amount was reduced approximately 15-fold compared to WT, suggesting that DSBs promote Zip1 S816 phosphorylation (Fig 6B, 6D and 6E) [44]. We conclude that there is a low background level of Zip1 S816 phosphorylation in the absence of DSBs, and that phosphorylation of this amino acid is induced above this background by meiotic DSBs.

Whether DSB formation is sufficient for Zip1 S816 phosphorylation was determined using an *sae2Δ/com1Δ* (SGD S000003143) diploid where the ends of the breaks are covalently attached to Spo11 and are not resected [45,46]. Phosphorylation of Zip1 S816 was observed in this mutant, suggesting that DNA processing is not necessary (Fig 6B). In *dmc1Δ* and *ndt80Δ* diploids, which arrest with unrepaired DSBs and dHJs, respectively [15,47], Zip1 protein was not degraded and p-S816 also persisted.

### Phosphorylation of Zip1 S816 Is Promoted by Mek1 Kinase Activity

The SILAC experiment showing that Zip1 S816-S818 phosphosites are potentially regulated by Mek1 is consistent with the induction of S816 phosphorylation by DSBs, since one consequence of making DSBs is activation of the kinase [6]. In fact, Zip1 S816 phosphorylation is reduced approximately 8-fold in a *mek1-K199R* diploid that encodes a catalytically inactive version of the kinase (Fig 6C, 6D and 6E) [48]. Mek1 activation occurs via the Mec1 and Tel1 checkpoint kinases, which phosphorylate the AE component Hop1 in response to DSBs, resulting in Mek1 recruitment to axes [7]. A 7-fold reduction in Zip1 S816 phosphorylation was observed in a *mec1Δ* strain, while a *tel1Δ* diploid appeared more like WT (Fig 6C, 6D and 6E). These results are similar to what was observed for phosphorylation of Hop1 by these kinases [6] and are consistent with Zip1 phosphorylation being promoted by activation of Mek1 via Hop1 phosphorylation. We conclude that Zip1 4S phosphorylation is induced upon DSB formation as a result of Mek1 activation and does not require the removal of Spo11 from the ends of the breaks or DSB resection.

### Zip1 Is a Direct Substrate of DDK

**Fig 7:**
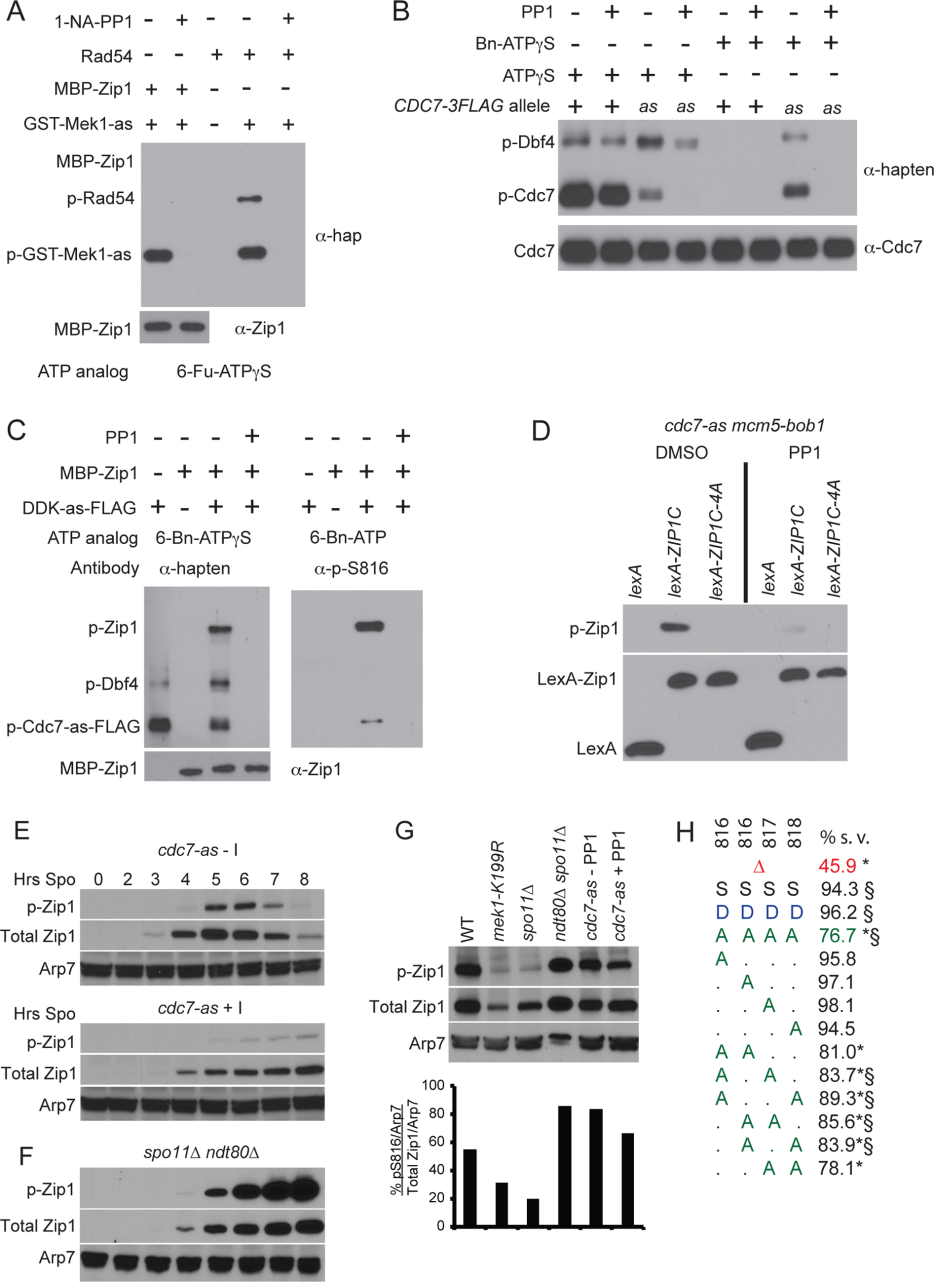
Phosphorylation of Zip1-S816 in vitro and in vivo. (A) Kinase assays using GST-Mek1-as (GST: Unitprot P0A9D2) partially purified from meiotic yeast cells and MBP-Zip1 (MBP: Unitprot P19642) or Rad54 as indicated with the ATP analog, 6-Fu-ATPγS. Phosphorylated proteins were detected using the semisynthetic epitope system with α-hapten (α-hap) antibodies [53]. The presence of MBP-Zip1 was confirmed using antibodies against total Zip1 (α-Zip1). The 1-NA-PP1 inhibitor concentration was 1 μM. (B) Kinase assays comparing autophosphorylation of DDK-FLAG and DDK-as-FLAG partially purified from meiotic yeast cells in the presence of ATPγS or the ATP analog, 6-benzyl (Bn)-ATPγS. The PPI inhibitor was used at a concentration of 20 μM. (C) DDK-as-FLAG kinase assays with MBP-Zip1. Left panel: 6-Bn-ATPγS was used with α-hapten antibodies to detect phosphorylated proteins by the semisynthetic epitope system. Right panel: 6-Bn-ATP was used with α -phospho-S816 antibodies to specifically detect Zip1 p-S816. (D) Zip1-S816 phosphorylation in vegetative cells with and without DDK-as kinase activity. The haploid *cdc7-as mcm5-bob1* strain, NH2309-6-3, was transformed with plasmids constitutively expressing *lexA*, *lexA-ZIP1C* ^*749-875*^, or *lexA-ZIP1C* ^*749-875*^ *-4A* and grown in the presence of either dimethylsulfoxide (DMSO) or 15 μM PP1. Extracts were probed with α-pS816 or α-lexA antibodies to detect Zip1 p-S816 and LexA, respectively. (E) Zip1 S816 phosphorylation in meiotic cells with and without DDK-as kinase activity. The *cdc7-as3-9myc* (called *cdc7-as* for short) diploid, NH2322, was transferred to Spo medium and the culture split in half at the 0 hour time point. To one half, DMSO was added (-I) and to the other a final concentration of 20 μM PP1 was added. Time points were collected and protein extracts assayed for the total amount of Zip1 as well as Zip1 p-S816. (F) Zip1 S816 phosphorylation in an *ndt80Δ spo11Δ* (NH2337) diploid. (G) Composite gel using extracts from time points with the highest amount of Zip1 from the indicated strains were run on the same gel and probed for total Zip1, p-S816, and Arp7. Quantification was performed as in Fig 6E. (H) Spore viability (% s.v.) of different *ZIP1* mutant alleles. *Δ* = *zip1Δ*; A = alanine, S and “.” = serine, D = aspartic acid. * and § = significantly different from WT and *zip1-4A*, respectively. See also S1 Table.

While Mek1 kinase activity promotes Zip1 S816 phosphorylation, this site does not match the Mek1 consensus (RXXT) determined both by screening peptide libraries and examination of in vivo Mek1 targets [11,49–51], suggesting that the regulation may be indirect. In vitro assays were therefore performed to see if Zip1 is a direct target of Mek1 using an analog-sensitive version of Mek1, GST-Mek1-as, and an analog of ATP, 6-furfuryl (Fu)-ATPγS [30]. Phosphorylation was detected using the semisynthetic epitope system, in which the kinase transfers a thiophosphate onto its target protein [52,53]. The thiophosphate is then alkylated with *p*-nitrobenzyl mesylate (PNBM) to create an epitope that is recognized by a commercially available antibody. Consistent with published results, autophosphorylation of GST-Mek1-as, as well as phosphorylation in *trans* of Rad54 (SGD S000003131), was observed, both of which were abolished by addition of the GST-Mek1-as inhibitor, 1-NA-PP1 (Fig 7A) [11]. No phosphorylation of recombinant MBP-Zip1 was detected, however, indicating that Zip1 is not a direct substrate of Mek1 in vitro (Fig 7A).

The four adjacent serines containing S816 represent potential phosphorylation sites for the highly conserved, essential kinase, DDK [54]. DDK is an acid-directed kinase that prefers to phosphorylate amino acids immediately upstream of a negative charge [28,55–57]. This charge can be provided either by priming phosphorylation or by negatively charged amino acids. To test whether DDK can directly phosphorylate Zip1, an in vitro kinase assay was developed using an analog-sensitive version of Cdc7, Cdc7-as [58]. *CDC7* or *cdc7-as* was fused to sequences encoding FLAG epitopes and the proteins partially purified from *ndt80Δ* -arrested cells. When incubated with ATPγS, autophosphorylation of Cdc7, as well as *trans* phosphorylation of Dbf4, was detected with DDK using the semisynthetic epitope system (Fig 7B). As expected, phosphorylation by DDK was unaffected by the addition of the DDK-as inhibitor, PP1 [58]. Cdc7-as-Dbf4 (DDK-as) was also able to use ATPγS, although not as efficiently as DDK (Fig 7B). Addition of PP1 to this reaction eliminated Cdc7-as autophosphorylation but not Dbf4 phosphorylation, indicating the presence of a contaminating kinase in the DDK-as preparation capable of modifying Dbf4. Specificity for DDK-as phosphorylation was achieved using the ATP analog, 6-benzyl-ATPγS (6-Bn-ATPγS) instead of ATPγS. In this situation, DDK-as, and not DDK, exhibited phosphorylation of Cdc7-as and Dbf4, and phosphorylation of both proteins was completely eliminated by the addition of PP1 (Fig 7B). DDK-as can therefore be used with 6-Bn-ATPγS to reveal direct targets of the kinase in vitro.

Unlike GST-Mek1-as, DDK-as directly phosphorylated MBP-Zip1 in vitro, and this phosphorylation was abolished by PP1 (Fig 7C, left). The semisynthetic epitope kinase assay detects phosphorylation anywhere on a protein and does not reveal specifically which amino acid(s) were phosphorylated. Kinase reactions were therefore performed with 6-Bn-ATP (which transfers phosphate instead of thio-phosphate), and the proteins were probed with p-S816 antibodies. Phosphorylated Zip1 S816 was observed, but not in the presence of inhibitor, confirming that Zip1 S816 is a direct target of DDK-as in vitro (Fig 7C, right).

To see whether DDK is capable of phosphorylating Zip1 S816 in vivo in mitotic cells, the Zip1 C terminus (amino acids 749–875) was fused to lexA (Unitprot P31080) (*lexA-ZIP1C*) and ectopically expressed in vegetative cells using the *ADH1* (SGD S000005446) promoter in a *cdc7-as mcm5-bob1* (SGD S000004264) strain. The *mcm5-bob1* mutation bypasses the essential function of *cdc7-as* in DNA replication initiation and therefore allows *cdc7-as* strains to grow vegetatively in the presence of PP1 [59,60]. In the absence of inhibitor, Zip1 p-S816 was detected with the WT *lexA-ZIP1C* plasmid, but not the *lexA-ZIP1C-4A* mutant (Fig 7D). Phosphorylation of S816 was reduced when DDK-as was inactivated by PP1, confirming that Zip1 S816 is an in vivo target of DDK-as. The low level of residual phosphorylation is presumably equivalent to the DSB-independent phosphorylation observed in *spo11Δ* meiotic experiments. The fact that DDK can phosphorylate Zip1 S816 in vegetative cells suggests that constraints on DDK phosphorylation of Zip1 are imposed specifically during meiosis.

Inhibiting DDK during meiosis is predicted to reduce Zip1 S816 phosphorylation because DDK is required for making DSBs, which in turn promote Zip1 S816 phosphorylation [58] (Fig 6). This prediction was partially borne out in that sporulating *cdc7-as* cells in the presence of PP1 resulted in a slight reduction in phosphorylation, but the decrease was not as great as that observed for *spo11Δ* (Figs 6, 7E, 7F and 7G). One difference between *spo11Δ* and *cdc7-as* + PP1 strains is that *spo11Δ* cells are able to progress into the meiotic divisions and degrade Zip1, while *cdc7-as* + PP1 cells are arrested prior to *NDT80* induction due to the requirement of DDK for *NDT80* transcription [61]. Zip1 S816 phosphorylation was therefore assayed in an *ndt80Δ spo11Δ* diploid, which should phenocopy both the DSB and meiotic progression defects of *cdc7-as* + PP1. Zip1 S816 phosphorylation was increased over four fold in the *ndt80Δ spo11Δ* diploid compared to *spo11Δ*, suggesting that phosphorylation of Zip1 S816 is no longer constrained at the *ndt80Δ* arrest. The fact that S816 phosphorylation is slightly reduced in the absence of DDK activity suggests that some of this phosphorylation is occurring due to DDK. However, the fact that phosphorylation is still present indicates either that the DDK inhibition was incomplete, or more likely, that there is a second kinase capable of phosphorylating this site independently of DDK. Our preferred hypothesis is that during normal meiosis, Zip1 phosphorylation is regulated by Mek1 and is mediated by DDK, but we cannot rule out the possibility that a second, yet-identified kinase also plays a role in Zip1 regulation.

There is no negatively charged amino acid immediately downstream of the Zip1 4S sequence (Fig 1D), suggesting that a priming negative charge is not necessary for DDK phosphorylation of recombinant Zip1. This appears to be true in vivo as well, as alanine substitutions of any one of the four serines supported WT spore viability (Fig 7H, S1 Table). Various combinations of double alanine substitutions reduced spore viability significantly from WT, but only the *zip1-S817A S818A* double mutant was as low as the level observed for the *zip1-4A* diploid. Interestingly, in TF proteins from other species, the predicted phosphoserines in the region conserved with Zip1 4S flank a negatively charged amino acid equivalent to the position of Zip1 S817 (Fig 1E). This is consistent with the hypothesis that a negatively charged patch created by phosphorylation in this region is a conserved requirement for TF function.

### *SGS1* and Zip1 4S Phosphorylation Are Both Required for Meiotic Progression

**Fig 8:**
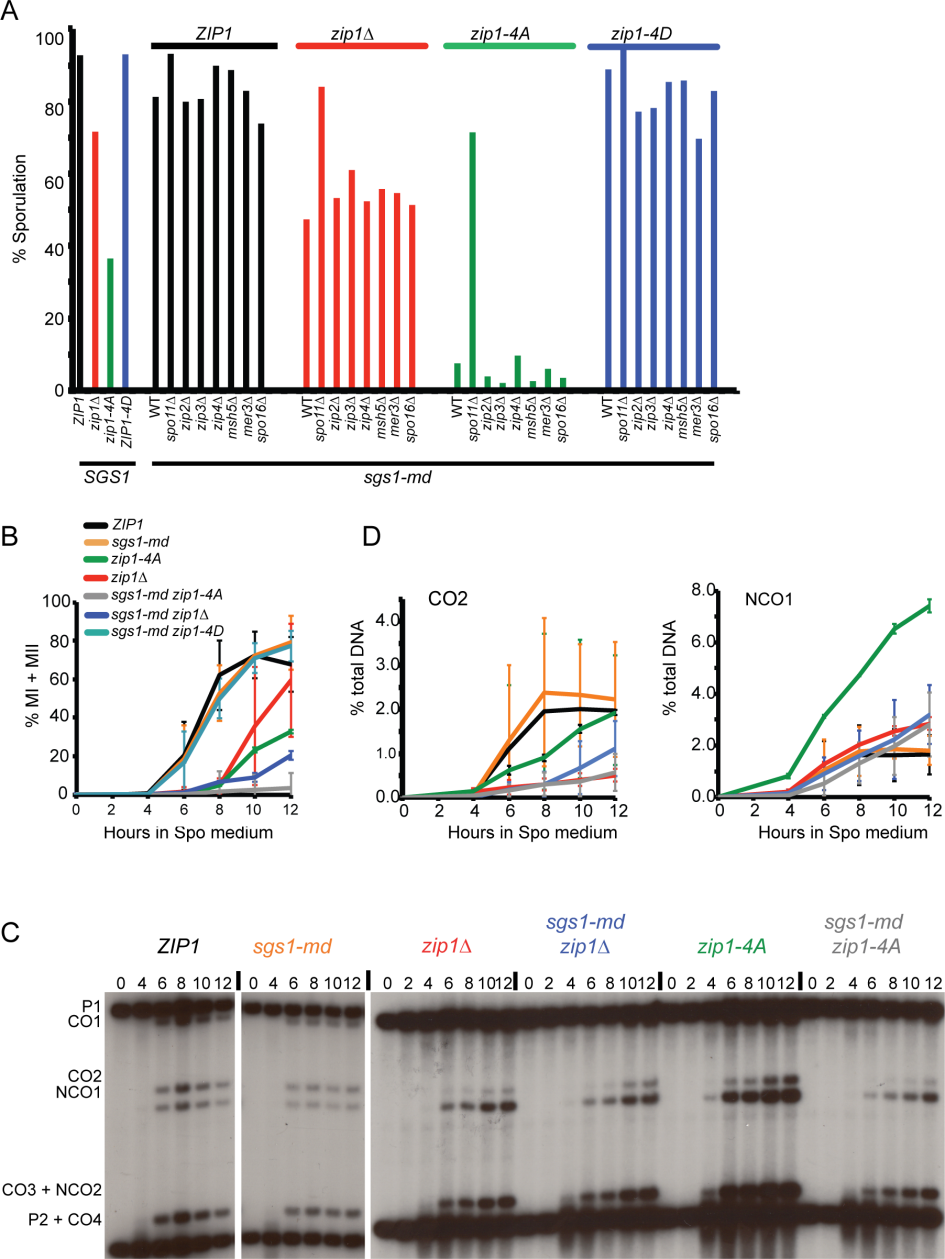
Meiotic phenotypes resulting from depleting *SGS1* in various *zip1* and *zmm* mutants. (A) Diploids of the indicated genotypes were sporulated for two days and the number of asci determined as in Fig 4 (For strain names see S1 Data). At least three independent transformants were assayed for each strain. (B) Meiotic progression of *ZIP1*, *sgs1-md*, *zip1Δ*, *zip1-4A*, *sgs1-md zip1Δ*, *sgs1-md zip1-4A*, and *sgs1-md zip1-4D* was monitored by DAPI staining of nuclei. The average values from different experiments were plotted with vertical lines representing the standard deviation. For *ZIP1*, *zip1Δ*, and *zip1-4D*, *n* = 3, for *sgs1-md* and *zip1-4A*, *n* = 2, for *sgs1-md zip1Δ* and *sgs1-md zip1-4A*, *n* = 4. The single mutant experiments are independent of the ones shown in Fig 2. (C) Physical analysis of COs and NCOs at the *HIS4*::*LEU2* hotspot. DNA was isolated at different time points and digested with XhoI/NgoMIV to detect restriction fragments representative of COs and NCOs. The *ZIP1* and *sgs1-md* panels are from nonadjacent lanes on the same gel, while the remaining four strains are from a different gel. (D) Quantification of CO2 and NCO1 fragments at the *HIS4-LEU2* hotspot. The average values from different experiments are plotted with vertical lines indicating the standard deviation. For *ZIP1*, *sgs1-md*, *zip1Δ*, and *zip1-4A*, *n* = 2. For *sgs1-md zip1Δ* and *sgs1-md zip1-4A*, *n* = 3.

Zmm proteins are proposed to stabilize recombination intermediates by protecting them from the action of the Sgs1 helicase [16]. The *sgs1-md* allele has *SGS1* under the control of the *CLB2* promoter that is not expressed in meiotic cells, thereby allowing selective depletion of Sgs1 during meiosis [62]. Depleting *SGS1* in the *zip1-4A* background produced an unexpected result: a large decrease in sporulation from 45.0% for *sgs1-md zip1Δ* to 8.5% for *sgs1-md zip1-4A* (Fig 8A). The failure to sporulate is due to meiotic prophase arrest, as *sgs1-md zip1-4A* cells do not undergo the meiotic divisions, remaining mononucleate and failing to generate MI and Meiosis II (MII) spindles measured by staining of nuclei and spindles with 4’, 6-diamidino-2-phenylindole (DAPI) and tubulin, respectively (Fig 8B and S4 Fig). The *sgs1-md zip1-4A* meiotic prophase arrest is due to the absence of Zip1 4S phosphorylation, as an *sgs1-md zip1-4D* strain went through the meiotic divisions more quickly and efficiently than *sgs1-md zip1-4A* (Fig 8B). The *zip1-4A* mutant alone reduced sporulation compared to WT in this background, but not to the low level observed in combination with *sgs1-md*. Therefore, *SGS1* prevents the meiotic prophase arrest resulting from failure to phosphorylate Zip1 4S.

Analysis of recombination products in the *sgs1-md* background revealed that the excess of NCOs observed in *zip1-4A* is dependent upon *SGS1*. Previously it has been observed that depletion of *SGS1* can partially suppress the CO defects of different *zmm* mutants to various extents [19,23,63]. While this phenotype was observed for the *sgs1-md zip1Δ* combination at the *HIS4-LEU2* hotspot, COs were reduced in the *sgs1-md zip1-4A* diploid (Fig 8C and 8D). This result is consistent with the idea that many of the DSBs in *sgs1-md zip1-4A* cells are unavailable for repair.

### Zip1 4S Phosphorylation Functions Upstream of Other *ZMM* Genes

The meiotic progression and sporulation defects observed for *sgs1-md zip1-4A*, but not *sgs1-md zip1Δ*, indicate that the presence of the Zip1-4A protein has a toxic gain of function phenotype in the absence of *SGS1*. One way to explain this phenotype would be if (1) Zip1 is recruited to a subset of DSBs where phosphorylation of Zip1 4S commits the breaks to repair by the *ZMM* pathway of interfering CO formation; (2) the absence of Zip1 4S phosphorylation blocks the break from being repaired, and (3) Sgs1 removes Zip1-4A from the breaks, thereby allowing them to be recycled and repaired via SDSA or the alternative Mus81-Mms4/Yen1 pathway. This model posits that the *sgs1-md zip1-4A* meiotic prophase arrest is due to unrepaired DSBs triggering the meiotic recombination checkpoint and is supported by the observation that preventing DSBs using *spo11Δ* restored sporulation to *sgs1-md zip1-4A* (Fig 8A).

Although the *ZMM* genes function in the pathway for producing interfering COs, the order in which they work has yet to be elucidated. The *zip1-4A* inhibition of sporulation in *sgs1-md* strains provides an assay by which to order *ZMM* genes relative to Zip1 4S phosphorylation. There are two ways that a *ZMM* gene could function prior to Zip1 4S phosphorylation. One possibility would be that a *ZMM* gene is required for Zip1-4S phosphorylation. In this case, the *sgs1-md zmmΔ* diploid would contain unphosphorylated Zip1 4S and should therefore phenocopy *sgs1-md zip1-4A* and fail to sporulate. This was not observed, however, as combining *sgs1-md* with a deletion of *ZIP2*, *ZIP3*, *ZIP4*, *MSH5*, *MER3*, or *SPO16* resulted in high levels of sporulation (Fig 8A). Consistent with this observation, Zip1 S816 phosphorylation was unaffected in *sgs1-md* diploids containing *mer3Δ*, *msh5Δ* or *zip3Δ* (S1B Fig). Another possibility is that a *ZMM* gene is required for Zip1 4S function (for example if it was needed to recruit Zip1 to the DSB). In this scenario, the absence of the *ZMM* gene should phenocopy the *spo11Δ zip1-4A sgs1-md* diploid and allow *sgs1-md zip1-4A zmmΔ* diploids to sporulate. This was not observed, either, as all of the *sgs1-md zip1-4A zmmΔ* diploids exhibited decreased sporulation (Fig 8A). A simple interpretation of these data is that these *ZMM* genes function after Zip1 4S phosphorylation.

## Discussion

It has recently become clear that whether a meiotic DSB is repaired as a CO or NCO is due primarily to the interplay between the “anti-CO” activities of helicases and the “pro-CO” activities of the functionally diverse set of Zmm proteins [16]. Yet, how this interplay might be modulated was unclear. Our discovery that phosphorylation of serines S815–818 in the Zip1 C terminus is required for the *ZMM* pathway of CO formation suggests a mechanism by which the CO/NCO decision can be regulated.

### Phosphorylation of the Zip1 C Terminus Initiates the *ZMM* Pathway of Interfering COs

The Zip1 protein contains N and C terminal globular domains flanking coiled-coil regions [12]. In the SC, TF proteins such as Zip1 form homo-oligomers in which the carboxy (C) termini are in contact with AEs, while the amino (N) termini interact within the central region [2]. While the Zip1 N terminus is unnecessary for synapsis [12], phosphorylation of serines 815–818 within the C terminus is required for SC formation. Failure to phosphorylate Zip1 S815–818 produces a canonical *zmm* phenotype. There is a delay in the formation of SEI and dHJ recombination intermediates. COs are reduced on small chromosomes, resulting in MI non-disjunction and decreased spore viability. Although CO defects are less obvious on larger chromosomes, CO interference is weakened or absent on both small and large chromosomes, indicating that *ZMM*-mediated COs are affected on all chromosomes. NCOs are increased in an *SGS1*-dependent manner, as expected if DSBs that cannot be repaired via the *ZMM* pathway are channeled by *SGS1* into pathways that generate NCOs either through SDSA or unbiased HJ resolution. In addition, the increased spore viability observed in *zip1-4A* relative to *zip1Δ* is dependent upon *MUS81*. Therefore, Zip1 4S phosphorylation is required to antagonize the anti-CO function of Sgs1.

### Zip1 4S Phosphorylation Functions after DSB Formation But Prior to Other *ZMM* Genes

**Fig 9:**
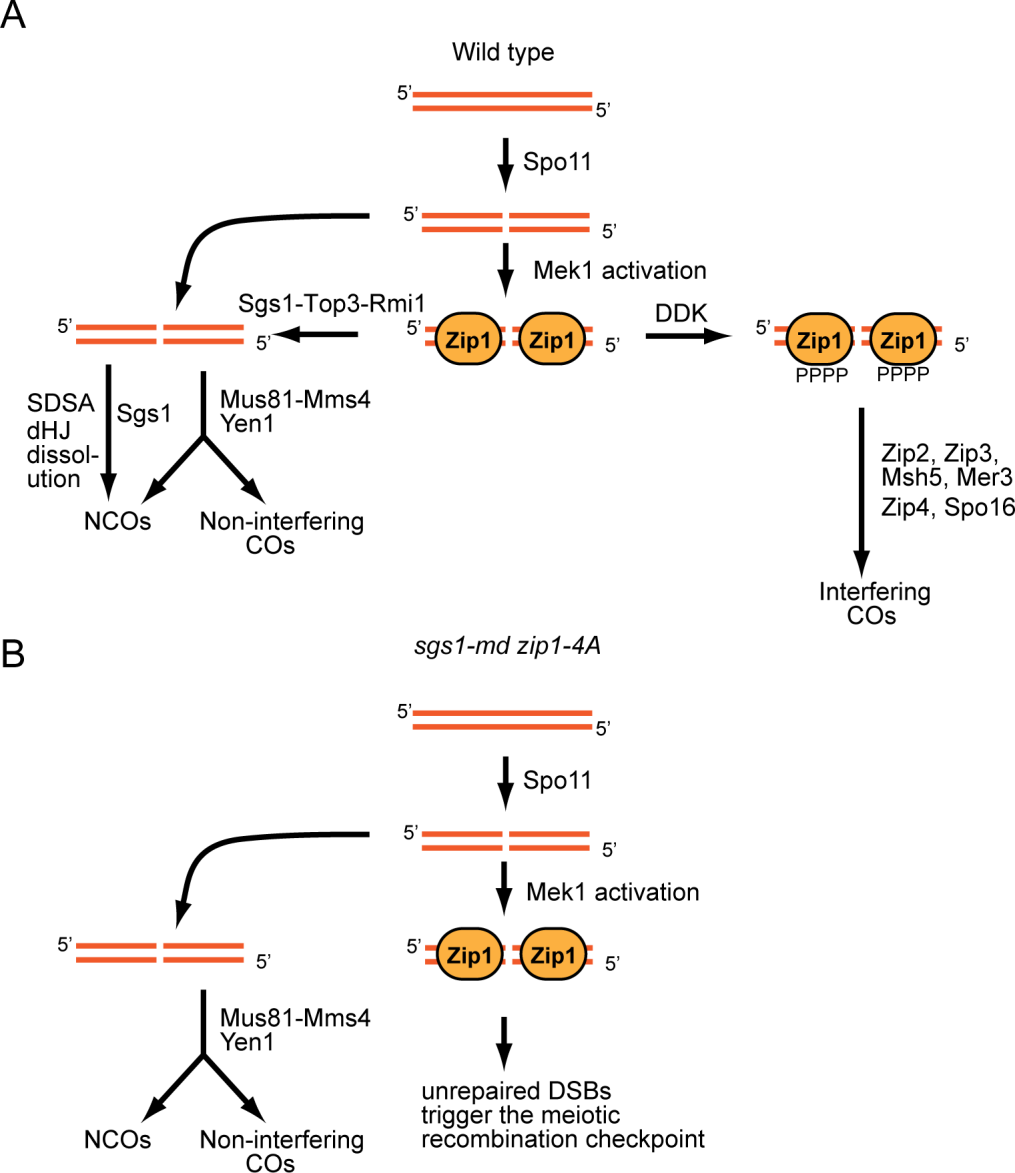
Model for the role of Zip1 4S phosphorylation is regulating COs. (A) Wild-type cells. STR stands for Sgs1-Top3-Rmi1. (B) *sgs1-md* cells.

In yeast, it has previously been hypothesized that the CO/NCO decision is made soon after DSBs are generated [13,24]. In WT cells, Zip1 phosphorylation coincides with the appearance of phosphorylated Hop1, an indirect marker of DSBs. Furthermore, Zip1 phosphorylation is promoted by Mek1 kinase activity, which is activated in response to DSBs [6,51] and reduced when DSBs are prevented by deletion of *SPO11*. We propose that Zip1 associates with DSBs (either directly or through interaction with other proteins) and that 4S phosphorylation then commits those breaks to the *ZMM* pathway (Fig 9A). In fact, association of Zip1 and other Zmm proteins at DSBs has recently been shown to be promoted by the 9-1-1 checkpoint clamp complex that binds to the single strand DNA-double strand DNA junctions of resected ends [64].

Epistasis analysis indicates that Zip1 4S phosphorylation functions prior to all of the other *ZMM* genes. Although this finding is at odds with cytological studies showing that *ZIP2* and *ZIP3* are required for the assembly of Zip1 along the SC, it is consistent with the fact that Zip3 localization specifically to DSB sites (measured by chromatin immunoprecipitation) is dependent upon *ZIP1* [2,39]. We propose that Zip1 has two temporally distinct functions with regard to SC formation. Its early function is to localize to DSBs where phosphorylation of the 4S region creates a patch of negative charges at the C terminus that promotes Zip1 interaction with downstream proteins, thereby committing the DSB to an interfering CO fate (Fig 9A). The resulting protein complex, which includes Zip2 and Zip3, is then necessary to nucleate Zip1 polymerization to allow synapsis.

Diploids lacking both *SGS1* and *ZIP1* are able to sporulate, whereas *sgs1-md zip1-4A* strains are not. Furthermore, the increased number of NCOs *in zip1-4A* is dependent upon *SGS1*. These results suggest that Sgs1 is able to remove Zip1-4A from DSBs, thereby allowing breaks to be repaired by alternative pathways. In *sgs1-md* strains, the DSBs associated with Zip1-4A cannot be repaired and trigger the meiotic recombination checkpoint (Fig 9B). We further hypothesize that there is competition for DSBs between Sgs1 and Zip1 and this competition can be biased towards the *ZMM* pathway by phosphorylation of Zip1. This provides a mechanism for influencing which pathway is preferred to implement CO homeostasis. For example, when DSBs are reduced, CO levels could be kept constant by increasing the fraction of DSBs containing phosphorylated Zip1.

### The requirement for Zip1 4S Phosphorylation for Interhomolog Engagement Obscures the CO Defect for *zip1-4A* on Large Chromosomes

In addition to their effect on CO regulation, *ZMM*s are required for synapsis and to mediate a type of IH engagement that results in feedback inhibition of Spo11 [13,14]. This is also true for Zip1 4S phosphorylation as *zip1-4A* mutants exhibit higher levels of DSBs that persist longer than WT. Interestingly, DSBs in *zip1-4A* are present at higher levels and for longer than *zip1Δ* as well. This suggests that, while *zip1Δ* cells eventually adapt to the lack of *ZMM*-mediated IH engagement and down-regulate Spo11, the presence of the Zip1-4A protein actively prevents this adaptation.

When IH engagement is defective, additional DSBs are introduced disproportionately onto larger chromosomes [14]. We propose that some of these extra DSBs are converted into COs via the alternative *MUS81*-dependent pathway and that the higher number of DSBs formed in *zip1-4A* on larger chromosomes consequently increases the number of COs, thereby providing an explanation for the higher spore viability of *zip1-4A* compared to *zip1Δ*. These results underscore the complexity in analyzing *zmm* mutant phenotypes. While *zip1Δ* and other *zmms* are commonly considered to be CO-defective, both *zip1Δ* and *zip1-4A* may exhibit WT (or greater) levels of COs depending on the chromosome that is being assayed. The fact that interference is reduced on all chromosomes, large and small, indicates that the *ZMM* pathway is not functioning on large chromosomes either, but the *ZMM* CO defect is obscured by the creation of COs along the *MUS81*-independent pathway that do not exhibit interference [35].

In addition to being required for recombination and synapsis, Zip1 also functions at centromeres. Prior to DSB formation, Zip1 promotes homology-independent centromere coupling, which is then regulated by Mec1 phosphorylation of S75 in the Zip1 N terminus [65,66]. Both *zip1-4A* and *zip1-4D* are functional for nonhomologous centromere coupling, as might be expected given that the 4S sequence is in the C terminal globular domain (S5 Fig). After SC disassembly, Zip1 remains at centromeres and helps promote segregation of nonexchange chromosomes [67,68]. While it is possible that the higher spore viability observed for *zip1-4A* compared to *zip1Δ* is due to this post-pachytene chromosome segregation function, this idea would not explain why the increased spore viability is dependent upon *MUS81* and therefore seems unlikely.

### C Terminal Phosphorylation of TF Proteins May Be a Conserved Mechanism for Regulating Recombination

Phosphorylation of Zip1 S816 is promoted by two very different kinases: the meiosis-specific kinase, Mek1, and the highly conserved cell cycle kinase, DDK. A number of experiments indicate that DDK1 is directly phosphorylating Zip1, while the requirement for Mek1 is indirect. First, S816 is not contained within a Mek1 consensus sequence (RXXT), making it unlikely to be a direct substrate. In contrast, the sequence of consecutive serines in the Zip1 C terminus provides a good target for the addition of multiple phosphates by DDK, which normally has a preference for phosphorylating amino acids immediately upstream of a negative charge [28,55–57]. Second, DDK-as, but not Mek1-as, phosphorylates recombinant Zip1 in vitro. The use of analog-sensitive kinases with the appropriate ATP analogs ensures that the phosphorylation observed in vitro is directly due to the analog-sensitive kinase. The specificity for DDK-as is further supported by the fact that Zip1 S816 phosphorylation is abolished in vitro by the DDK-as inhibitor, PP1. Third, the bulk of S816 phosphorylation observed in vegetative cells ectopically expressing the Zip1 C terminus is dependent upon DDK-as kinase activity, confirming that this reaction occurs in vivo, as well as in vitro.

Phosphorylation of Zip1 S816 is complex, however, as noted by the surprising observation that *ndt80Δ spo11Δ*-arrested cells exhibited high levels of Zip1 S816 phosphorylation in the absence of DSBs, while phosphorylation was reduced ~15-fold in *NDT80 spo11Δ* diploids. Like *spo11Δ ndt80Δ*, inactivation of DDK-as in meiosis prevents DSBs and meiotic progression [57,60]. The fact that S816 phosphorylation was not abolished but only partially reduced in these cells, even though DDK-as was inactive, indicates a second kinase can phosphorylate S816 when the cells are arrested. One possibility is that Zip1 phosphorylation simply correlates with the time that cells spend in meiotic prophase—so that mutants that cause delays in meiotic progression such as *sae2Δ*, *dmc1Δ*, and *ndt80Δ* allow the phosphorylated form of the protein to accumulate. An alternative explanation is that the *ndt80Δ* arrest creates a situation in which the WT regulation of Zip1 phosphorylation is no longer working. It may be that, given enough time, DDK and/or the second kinase can phosphorylate Zip1 everywhere along the chromosome. The discovery of unregulated Zip1 S816 phosphorylation may explain the surprising observation that in *mek1Δ ndt80Δ* diploids, IH JMs that are suppressed early in meiosis (as expected due to the lack of IH bias) accumulate at the *ndt80Δ* arrest [69]. This result also raises the question as to why Mek1 regulation of S816 was observed in the SILAC experiment in which cells were arrested using *ndt80Δ*. One potential explanation is that phosphopeptides identified in the SILAC experiment were derived from chromatin-associated proteins, while the western blots probed with the α-pS816 antibodies were from whole cell extracts, which do not provide any information as to location of the Zip1 that is getting phosphorylated.

DDK is an essential protein kinase with an evolutionarily conserved role in the initiation of replication [70]. Given that DDK directly phosphorylates Zip1 S816 and is responsible for the bulk of phosphorylation observed when the Zip1 C terminus is ectopically expressed in vegetative cells, a reasonable hypothesis is that DDK has a role in regulating CO formation in yeast, raising the question as to whether this role is also evolutionarily conserved. In fact, the TF proteins from mammals and zebrafish contain homology to the yeast 4S region. This conservation is especially striking because overall homology between TF proteins is low [2]. In addition, multiple residues within the 4S region are predicted to be phosphorylated in mammals and zebrafish. This, along with the fact that many of the yeast Zmm proteins have orthologs in mammals, supports the idea that this mechanism for CO regulation is conserved [71]. This mechanism may not be universal, however, as no homology was found in the TF proteins from plants, although plants appear to contain at least some *ZMM* genes (e.g., [72]). In addition, TF proteins from both *D*. *melanogaster* and *C*. *elegans* do not contain the 4S region. These organisms do not use recombination to initiate synapsis and therefore may not need the homolog interaction function of TF proteins mediated by phosphorylation [2].

### *MEK1*-Dependent Phosphorylation of Zip1 4S Provides a Link between IH Bias and the CO/NCO Decision

DSBs trigger the localized activation of Mek1 on the axis. A role for Mek1 in promoting IH bias is well-established as inactivation of Mek1 results in IS repair [9,43]. This work shows that Mek1 regulates IH CO formation as well, by promoting phosphorylation of the C terminus of Zip1. Mek1 can therefore be considered a “master regulator” of meiotic recombination in that it phosphorylates substrates that both suppress IS recombination while at the same time promoting interfering CO formation.

The observation that Zip1 S816 is phosphorylated in vegetative cells but is induced by Mek1 and DSBs during WT meiosis suggests that Zip1 phosphorylation is constrained in meiotic cells and that Mek1 kinase activity overcomes this constraint. How Mek1 does this remains to be established. DSB formation is timed to occur after DNA replication [1]. DDK phosphorylation of Mer2 (SGD S000003782) promotes the recruitment of Spo11 to axes where DSBs occur. Recent work has shown that DDK travels with the replication fork, phosphorylating Mer2 and then moving on [73]. Since Zip1 is not phosphorylated until after DSBs have been made, it seems unlikely that replication fork-associated DDK is responsible. Therefore, one possibility is that Mek1 phosphorylation of itself or some other protein results in the recruitment of DDK (or potentially the alternative kinase) to DSBs, either by creating a “landing pad” for the kinase or by changing the chromosomal architecture to make breaks more accessible. For example, Mek1 has been proposed to antagonize Rec8-mediated cohesion locally around DSBs to release an “homology searching tentacle”, which could then attract the kinase [9]. We propose that Mek1 kinase activity is the link by which IH bias and IH CO formation are coordinated to ensure proper chromosome segregation during meiosis.

## Materials and Methods

### Yeast Strains

The genotypes of all strains used in this work can be found in S2 Table. With the exception of experiments shown in S3 Fig and S5 Fig, all strains were derived from the SK1 background. Genes were deleted by polymerase chain reaction (PCR)-based methods using the *kanMX6*, *natMX4*, or *hphMX4* markers that confer resistance to G418, nourseothricin, and Hygromycin B, respectively [74–76]. PCR fragments using *Kluyveromyces lactis URA3* gene as a selectable marker were also used to make precise gene deletions (pKlU, A. Neiman, Stony Brook University). Unless stated otherwise, all deletions were confirmed using colony PCR.

The *mek1-as ndt80Δ lys4Δ arg4* diploid, NH2221, was constructed in several steps. First, *LYS4* (SGD S000002642) was deleted in the *dmc1Δ mek1Δ* haploid, DKB187 mek1Δ. This haploid was crossed to S2683 [77] and the diploid dissected to get haploids of opposite mating type containing *dmc1Δ*::*LEU2*, *mek1Δ*::*kanMX6*, *lys4Δ*::*hphMX4*, and *arg4-Nsp* (SGD S000001060) (*LEU2*: SGD S000000523). The *mek1Δ*::*kanMX6* allele was exchanged for *mek1-as* by two-step gene replacement using the *mek1-as URA3* plasmid, pJR2 [78,79]. The haploids were then converted to *DMC1* by integration of the *URA3 DMC1* plasmid, pNH301 [10]. After deletion of *NDT80* with *natMX4*, the haploids were mated to make the NH2221 diploid.

To enable integration of the *ZIP1 TRP1* (SGD S000002414) plasmid, p382 (generously provided by A. Hochwagen, New York University), the first 222 bp of the *TRP1* open reading frame (ORF) were deleted from NHY1210 and NHY1215. The presence of the deletion was confirmed by failure to grow on SD-Trp medium. *ZIP1* was then deleted with various markers, and the two haploids mated to make diploids. The p382 plasmid was targeted to integrate into the 3’ end of the *TRP1* gene by digestion with BsgI. All transformants were checked by PCR using internal *ZIP1* primers to confirm the presence of the plasmid. The *sgs1-md* allele was introduced into the NHY1210 zip1 trp1 and NHY1215 zip1 trp1 haploids by replacing the *SGS1* promoter with the *GAL1* promoter using *kanMX6* [75]. *SPO11*, *SAE2* and various *ZMM* genes were then replaced with the *K*. *lactis URA3* gene. Various *zip1* alleles were introduced by integration of plasmids as described above into each haploid and mated to generate homozygous diploids.

NH2241 containing various alleles of *ZIP1* was constructed by first deleting *ZIP1* with *natMX4* from NHY942 and NHY943, the haploid parents of NHY957 [35]. The 222 bp *trp1-5’Δ* deletion was then introduced into NHY943 zip1 using *hphMX4*. This haploid was transformed with p382-4A (*zip1-4A*), p382-4D (*ZIP1-4D*), or pRS304 (vector) digested with BsgI to target integration to the 3’ end of *TRP1*. These haploids were then crossed to NHY942 zip1.

*CDC7* and *cdc7-as* were tagged with three copies of the FLAG epitope using pFA6a-3FLAG-kanMX6 [80] in S2683 and RKY1145 containing *ndt80Δ*::*hphMX4*. The haploids were then mated to make NH2082 and NNH2072.

BR5892-8A::pB211 is a *MAT* **a**/*MATα* haploid constructed by transformation of BR5892-8A with pB211 digested with NcoI to target integration to *thr1* [81]. NH2247 was generated by crossing *zip1*::*URA3 CTF19-myc-kanMX6* (*CTF19*: SGD S000005939) haploid to an isogenic *zip1Δ*::*LEU2* strain, dissecting and selecting segregants with all three alleles. These haploids were then crossed to make the homozygous diploid.

The *cdc7-as-9myc* diploid, NH2322, was made by first introducing the *cdc7-as3-9myc* allele into the NHY1210 and NHY1215 haploids [78] by two-step gene replacement [79]. The *URA3 cdc7-as3-9myc*-integrating plasmid, pJO36-as3 [58], digested with EcoRI to target integration downstream of the *CDC7* open reading frame, was used for the transformation. Ura^+^ transformants were then grown in nonselective medium, and plasmid pop-outs were selected using 5-fluorotic acid (5-FOA). FOA^r^ colonies that retained the *cdc7-as3-9myc allele* were assayed for the failure to grow in the presence of the 20 μM [4-amino-1-tert-butyl-3-(*p*-methylyphenyl)pyrazolo [3,4-d]pyrimindine] (PP1) inhibitor, and the *cdc7-as3-9myc* haploids were mated to make the diploid. The *cdc7-as mcm5-bob1 trp1Δ* strain was constructed in multiple steps. First, one of the *cdc7-as* alleles was deleted with *kanMX6* in the *cdc7-as mcm5-bob1* homozygous diploid, NH661 [60]. Second, the 222 bp *TRP1* 5’ deletion was created using *hphMX4*, and the resulting doubly heterozygous diploids sporulated and tetrads dissected to obtain a *cdc7-as mcm5-bob1 trp1-5’Δ*::*hphMX4* segregant called NH2309-6-3. Various *ZIP1* alleles were then introduced by integration of p382 or its derivatives.

### Plasmids

Mutations were introduced into p382 and pBTM116-ZIP1C^750-875^ (below) by site-directed mutagenesis (QuikChange kit, Stratagene) and confirmed by DNA sequencing by the Stony Brook University DNA Sequencing Facility. All alleles were sequenced in their entirety to ensure that no additional mutations were introduced. To ectopically express the C terminus of Zip1 in vegetative cells, a 383 bp fragment containing codons 749–875 was amplified by PCR. A BamHI site was engineered immediately upstream of *ZIP1* codon 750 in frame with *lexA* in pBTM116 [82] with a PstI site located immediately after the *ZIP1* stop codon. Ligation of the BamHI/PstI *ZIP1C* fragment into BamHI/PstI digested pBTM116 created pBTM116-ZIP1C-WT. The S815A S816A S817A S818 mutations were then introduced by site-directed mutagenesis. The pXC6 plasmid containing *ADE2 mek1-K199R* was constructed by subcloning a 2.9 kb NotI/SalI fragment from pLP34 [48] into NotI/Sal-digested pRS302. Digestion of pXC6 with StuI targets integration into *ade2*. pLT11 and pLW20 contain *HOP1 URA3* and *MEK1 ADE2*, respectively [43]. For *MBP-ZIP1* expression and purification, a 2.6 kb fragment of *ZIP1* was amplified by PCR in which XbaI and HindIII restriction sites were engineered immediately upstream of the ATG or downstream of the stop codon, respectively. This fragment was then cloned into pMAL-TEV (provided by Patrick Sung, Yale University) to fuse *ZIP1* to the gene encoding Maltose-Binding Protein (MBP) with a TEV cleavage site engineered between MBP and Zip1 to make pMAL-TEV-ZIP1-WT. pLW3 is a high copy number 2μ plasmid containing *URA3* and *GST-mek1-as* [30].

### Meiotic Time Courses

Cells were sporulated at 30°C as described in [53] with the exception of the time courses used for the physical analyses in Fig 2, which used 0.5% potassium acetate instead of 2% potassium as the Spo medium. Physical analysis of the *HIS4/LEU2* hotspot was performed as described in [24]. Chromosome spreads were performed as described in [83]. All primary antibodies were diluted 1:100. The Zip1 antibodies used for cytology were provided by A. MacQueen (Wesleyan) (BR strains) or S. Keeney (Sloan-Kettering Cancer Research Center) (SK1 strains). Meiotic progression was analyzed by staining cells with DAPI and using fluorescent microscopy to count the number of mononucleate, binucleate (MI), and tetranucleate (MII) cells. Two hundred cells were counted for each time point of each strain in each replicate.

### Identification of Phosphorylation Sites on Zip1

SILAC experiments were performed as described in [29] by growing the *mek1-as ndt80Δ* diploid, NH2221, in synthetic medium containing either arginine/lysine or arginine/lysine with stable heavy isotopes of ^13^C and ^15^N and then transferring the cells to 2% potassium acetate for 14 h to allow cells to arrest at pachytene [29]. 1-NA-PP1 was added to a final concentration of 1 μM to the heavy culture for 20 min to inactivate Mek1-as. Phosphopeptides were enriched and analyzed as described in [29].

### Protein Alignment and Phosphorylation Scores

Global alignments were performed in JalView (http://www.jalview.org) [84] version 2.8.2 using ClustalO [85] with default settings and viewed with a conservation threshold of 50%. Sequences were obtained from the NCBI Protein database (www.ncbi.nlm.nih.gov/protein/). TF homologs of *S*. *cerevisiae* Zip1 (Accession: AAA35239.1) are: *Homo sapiens* SYCP1 (Accession: NP_001269470.1), *Mus musculus* SCP-1 (Accession: NP_035646.2*)*, *Rattus norvegicus* SCP-1 (Accession: Q03410.2*)*, and *Danio rerio* SCP-1 (Accession: NP_001112366.1). Phosphorylation scores were calculated using NetPhos v2.0 (http://www.cbs.dtu.dk/services/NetPhos/) [32].

### Western Blots and Phosphatase Experiments

Protein extracts were prepared using the tri-chloroacetic acid (TCA) method as described by [65]. For phosphatase experiments, a modification of a protocol from Pedro San-Segundo was used [86]. 60 μl of TCA extracts in 1 X Laemmli sample buffer were diluted ~1:10 with 525 μl 1 X PMP buffer (50 mM HEPES, pH 7.5, 100 mM NaCl, 2 mM dithiolthreitol) from which 195 μl was aliquoted into two tubes. Alkaline phosphatase (5 μl) (Roche, #11097075001) was added to one tube, and 5 μl 1 X PMP buffer was added to the other tube. After incubation at 30°C for 30 min, the samples were put on ice and reprecipitated with TCA prior to fractionation by SDS-polyacrylamide gel electrophoresis and immunoblot analysis. After incubation with the appropriate secondary antibodies coupled to horse radish peroxidase (HRP—see below), the blots were developed using the Pierce ECL2 western blotting substrate (Thermo Scientific) and exposed to film.

To compare the fraction of S816 phosphorylated Zip1 in different strains, two sets of identical samples were run on the same gel and blotted to a membrane. The membranes were then cut vertically to separate the two sets and then horizontally to separate the Zip1 proteins from Arp7. The top half of each blot was probed with either total Zip1 or α-pS816 antibodies and the bottom halves with α-Arp7 antibodies. Quantification was performed by scanning the films using a Canon LiDE 110 scanner and analyzing the bands using ImageQuant TL 7.0 software. Both total Zip1 and pS816 signals were normalized by the amount of Arp7 loaded in the same lane, and the percent of phosphorylated Zip1 was calculated by pS816/Arp7^pS816^/Total Zip1/Arp7^Total Zip1^ *100.

### Antibodies

Antibodies specific for phosphorylated Zip1 S816 were generated by injecting a rabbit with the peptide, Ac-CDEFDLS(pS)SSND-amide (Covance Research Products). To isolate p-S816 antibodies, the serum was first passed over a column containing the unphosphorylated peptide. Antibodies that bound to the column (called 1907) were eluted and used to detect Zip1 lacking S816 phosphorylation. The flow through was loaded onto a column containing the phosphorylated peptide. These Zip1 phospho-S816-specific antibodies (called 1907-p) were eluted and stored at −80°C. The 1907 and 1907-p antibodies were used at 1:250 and 1:2,000 dilution, respectively.

Anti-Hop1 antibodies were used as described in [48]. Ndt80 antibodies (a gift from Michael Lichten) were used at a 1:15,000 dilution [87]. Arp7 polyclonal goat antibodies (Santa Cruz, SC-8960) were used at a dilution of 1:10,000 as a loading control. The secondary antibody was a 1:10,000 dilution of donkey α-goat IgG-HRP (Santa Cruz, SC-2020). For the BR chromosome spreads, 9E10 (myc antibodies), α-Red1 [30] and α-Zip1 (provided by A. MacQueen, Wesleyan University) antibodies were used at a 1:100 dilution, followed by Alex-fluor 488 goat α-rabbit secondary antibodies (Life Technologies, 1:100 dilution). For the SK1 chromosome spreads, an α-Zip1 antibody supplied by S. Keeney (Sloan-Kettering Cancer Research Center) was used. The α-hapten antibody used for the semisynthetic epitope method was purchased from Epitomics (#2686–1). Cdc7 protein was detected using goat α-Cdc7 antibodies (Santa Cruz, SC-11964) at a 1:250 dilution for the primary antibody. Total Zip1 was determined using a 1:3,000 dilution of a polyclonal rabbit antibody generated against amino acids 576–875 (Santa Cruz, SC-33733). A goat α-rabbit IgG-HRP secondary antibody (Santa Cruz, SC-2004) was then used at a dilution of 1:50,000.

### Protein Purification

GST-Mek1-as was partially purified from NH520/pLW3 as described in [53]. Cdc7-3FLAG and Cdc7-as-3-FLAG were partially purified out of the *ndt80Δ* diploids, NH2082 and NH2072, respectively, as described in [88]. Eight liters of cells incubated for 8 h in Spo medium at 30ºC were used to make protein extracts, which were then frozen in 10 ml aliquots. One 10 ml aliquot was used for the FLAG antibody purification. The Rad54 protein was purified as described in [89] and was generously supplied by Patrick Sung.

### Purification of MBP-Zip1

For purification of MBP-Zip1, pMAL-TEV-ZIP1-WT was transformed into the *E*. *coli* strain Rosetta 2 (EMD Millipore). An overnight culture was diluted into 4 L 2 X LB medium and grown to an OD_600_ of 0.8. Isopropyl β-D-1-thiogalactopyranoside was added to a final concentration of 0.2 mM and the cells incubated overnight at 16°C. Approximately 20 g of cell pellet was obtained from 4 L culture. The pellet was lysed in 100 mL of K buffer (20 mM KH_2_PO_4_, pH 7.4, 10% glycerol, 0.5 mM EDTA, 0.01% Igepal and 1 mM DTT) containing a cocktail of protease inhibitors (aprotinin, chymostatin, leupeptin, and pepstatin A at 5 μg/ml each and phenylmethylsulfonyl fluoride at 1 mM) and 500 mM KCl. The lysate was cleared by ultracentrifugation (100,000 X *g* for 45 min) and then incubated with 1 mL of Amylose resin (New England Biolabs) for 2 h. The matrix was washed three times with 20 mL of K buffer containing 500 mM KCl and 0.1% Igepal before MBP-Zip1 protein was eluted with 5 mL of K buffer containing 500 mM KCl and 10 mM Maltose. The eluate was further concentrated and filter dialyzed against K buffer plus 500 mM KCl. The purified MBP-Zip1 protein was frozen in liquid nitrogen and stored at −80°C as small aliquots.

### In Vitro Kinase Assays

For the GST-Mek1-as kinase assays using the semisynthetic epitope system, 24 μl reactions contained 263 nM GST-Mek1-as, 21 mM MgCl_2_, 83 μM ATP, and 150 nM of MBP-Zip1 or Rad54 [53]. Either 1 μl of DMSO or 1 μl 240 μM 1-NA-PP1 [1-(1,1-Dimethylethyl)-3-1(1-napththalenyl)-1H-pyrazolo[3, 4-d]pyrimidin-4-amine] (final concentration is 1 μM; Tocris #3063) was added and the reactions incubated at room temperature for 5 min. The analog, 6-Fu-ATPγS (BioLog #F008), was added to a final concentration of 100 μM to each reaction, which were then incubated for 30 min at 30°C. To detect thiophosphorylated proteins, PNBM (Epitomics #3700–1) was added to a final concentration of 2.5 mM, and the reactions were incubated at room temperature for 2 h. An equal volume of 2 X protein sample buffer was added to each tube and the proteins denatured by incubation for 5 min at 95°C. One-eighth of each reaction was fractionated on 7.5% SDS-polyacrylamide gels and transferred to PVDF membrane. Phosphorylation was detected by probing the immunoblot with a 1:10,000 dilution of the thiophosphate ester rabbit monoclonal antibody (called the α-hapten antibody for short; Epitomics, #2686–1) and detected using a 1:10,000 dilution of goat α-rabbit secondary antibody from Santa Cruz. For the Cdc7-3FLAG and Cdc7-as-3FLAG kinase assays using the semisynthetic epitope system, reactions contained 50 nM Cdc7-3FLAG or Cdc7-as-3FLAG, 50 mM Tris-HCl, pH 7.5, 10 mM MgCl_2_, 1 mM dithiolthreitol, 100 μM PMSF, 50 μM ATP, and 1 mM GTP, to which 150 nM of MBP-Zip1 was added. The remaining protocol was the same as for the GST-Mek1-as assays except that PP1 (generously provided by Kevan Shokat) was used as the inhibitor at a final concentration of 20 μM, and 6-BnATPγS (BioLog B072) was used as the ATP analog. To monitor Cdc7 phosphorylation of Zip1 S816, 6-Bn-ATP (BioLog B024) was used, the PNBM step was omitted, and the immunoblot was probed with a 1:2,000 dilution of the Zip1 phospho S816-specific antibody.

### Spo11 Oligonucleotide Analysis

Diploids were made fresh by mating haploids of the appropriate genotypes immediately prior to each experiment. Extracts for Spo11 oligo time courses were prepared as in [90] with the following modification: immunoprecipitations utilized 10 μl IgG Sepharose Fast Flow beads (GE) per 0.2 ml of extract. Immunoprecipitations were then performed exactly as in [14].

## Supporting Information

S1 Accession numbers List of the accession numbers for different genes and proteins described in the manuscript. (DOCX)

S1 Data Excel spreadsheet containing, in separate sheets, the underlying numerical data and statistical analyses for figure panels 2A, 2C, 2E, 2G, 3B, 4A, 4B and 4E, 4C, 4D, 4F, 5D, 6E, 7G, 8A, 8B, 8D, S2C, S3B, S4B, S5A, and S5B. (XLSX)

S1 Fig Biochemical analysis of Zip1 steady state protein levels and Zip1 S816 phosphorylation in different mutants. A. Time courses were performed at 30°C. Isogenic diploids containing either *zip1Δ* (NH2297::pRS304), *ZIP1* (NH2297::p382), *zip1-4A* (NH2297::p382-4A), or *ZIP1/zip1-4A* (NH2297::p382::p382-4A) were sporulated, and protein extracts were generated for the indicated time points. Equal amounts of extract were loaded for each time point for each strain. The gels were cut in half and the top half was probed with antibodies that recognize total Zip1, while the bottom half was probed with Arp7 as a loading control. B. Zip1 S816 phosphorylation in *sgs1-md* diploids containing different *zmm* mutants. Protein extracts from time courses from the *sgs1-md* diploids containing either *mer3Δ* (NH2267::p382)), *msh5Δ* (NH2265::p382), or *zip3Δ* (NH2243::p382) were probed with either the pS816 phospho-specific antibody or the total Zip1 antibody. The Arp7 loading control was performed as described in Panel A.
(TIF)

S2 Fig COs and JMs in various *zip1* mutants. A. Upper panel: Map of restriction site polymorphisms at the *HIS4*::*LEU2* meiotic DSB hotspot. Lower panel: Informative restriction fragments following digest with (i) XhoI or (ii) double digest with XhoI plus NgoMIV. B. Schematic representation of joint molecules detected by two-dimensional gel electrophoresis following digestion with XhoI. Asterisk: Only one of the four possible SEI species is drawn in detail (see [24]).C. Quantification of COs from two independent time courses. Error bars represent the range. D. One-dimensional Southern blot analysis of COs at the *HIS4*::*LEU2* hotspot for the time course shown in Fig 2. DNA was digested with XhoI. E. Complete two-dimensional Southern blot analysis to monitor JMs, excerpts of which are shown in Fig 2F.
(TIF)

S3 Fig SC formation in various *zip1* mutants. (A) Diploids from the BR background were incubated in Spo medium, and spread chromosomes were stained with anti-Red1 antibodies to visualize AEs and anti-myc antibodies to visualize the central element protein, Ecm11-myc. Red1-positive spreads were separated into categories: 1) lacking Ecm11, 2) dotty—exhibiting Ecm11 foci, 3) partial—exhibiting stretches of SC, or 4) full—exhibiting nearly complete or complete synapsis. (B) Quantification of the classes shown in Panel A. 100 spreads (*zip1-4A*) and 200 spreads (*ZIP1*, *ZIP1-4D*) were counted for each time point. (C) Comparison of Zip1 and Ecm11 staining in *ZIP1* and *zip1-4A* chromosome spreads.
(TIF)

S4 Fig Meiotic progression measured by spindle morphology in WT and *zip1-4A sgs1-md* diploids. Cells from the time course in Fig 8 for WT and *zip1-4A sgs1-md* stained with tubulin antibodies. A. Representative pictures of cells at different stages of meiosis with the indicated spindle morphology. B. Quantitation of different spindle types in the two mutants. No tubulin staining was observed at the 0, 2, and 4 hr time points in either strain. While the WT cells progressed through both MI and MII, >90% of the *zip1-4A sgs1-md* cells either had very short spindles indicative of pachytene or aberrant spindles. Therefore, these cells were arrested prior to MI.
(TIF)

S5 Fig Nonhomologous and homologous centromere associations in various *zip1* mutants. A. Nonhomologous centromere associations: The *MAT* **a** */MATα CTF19-MYC zip1Δ* haploid, BR5892-8A:pB211, was transformed with either *ZIP1* (p382), *ZIP1-4D* (p382-4D), *zip1-4A* (p382-4A), or pRS304 (vector). Twenty hours after transfer to Spo medium at 30°C, chromosomes were spread and stained with anti-myc (9E10) and anti-Red1 antibodies [30]. Spreads with substantial Red1 staining, indicating mid-prophase nuclei, were scored for the number of Ctf19 foci. Because haploids contain 16 nonhomologous chromosomes, pairing of nonhomologous centromeres should result in eight foci, as is observed for *ZIP1* [65,66]. In contrast, *zip1Δ* exhibits closer to the 16 foci expected if centromeres are unpaired. Both *zip1-4D* and zip1-*4A* appear to be fully functional for nonhomologous centromere association. B. Homologous centromere associations: Chromosomes from *CTF19-myc zip Δ* (NH2247) carrying various *ZIP1* alleles (*ZIP1*, *zip1D*, *zip1-4D* and *zip1-4A*) were spread 20 h after transfer to Spo medium and stained with anti-myc and anti-Red1 antibodies. Nuclear spreads with linear Red1 staining, indicating mid-prophase, were scored for the number of Ctf19 foci. Like *zip4Δ*, *zip2Δ* diploids exhibit ~16 foci since the chromosomes are homologously aligned and their centromeres are associated via Zip1 [66]. The *zip1Δ zip2Δ* mutant forms ~24 foci, consistent with the requirement for *ZIP1* to hold homologous centromeres together. This number is less than the 32 foci expected if all chromosomes are unassociated, presumably due to centromere proximal axial associations. *zip1-4D zip2Δ*, and *zip1-4A zip2Δ* exhibit Ctf19 focus numbers similar *to zip2Δ*, indicating these alleles are functional for homologous centromere association during prophase.
(TIF)

S1 Table Spore viability of various *zip1* mutants. (DOCX)

S2 Table *S*. *cerevisiae* strains. (DOCX)

## Abbreviations

AE: axial element
AP: alkaline phosphatase
BLM: Bloom syndrome RecQ helicase
CO: crossover, DAPI, 4’, 6-diamidino-2-phenylindole
DDK: Dbf4-dependent kinase
dHJ: double Holliday junction
DMSO: dimethylsulfoxide
DSB: double-strand break
IH: interhomolog
IS: intersister
JM: joint molecule
L/H: light:heavy
MI: Meiosis I
MII: Meiosis II
MS: mass spectrometry
NCO: noncrossover
NM: nonmating
NPD: nonparental ditype
PC: polycomplex
PD: parental ditype
PNBM: *p*-nitrobenzyl mesylate
SC: synaptonemal complex
SDSA: synthesis-dependent strand annealing
SEI: single end invasion
SILAC: stable isotope labeling of amino acids in cell culture
TF: transverse filament
TT: tetratype
WT: wild-type
