# Supplementary material for: Phosphorylation of the Synaptonemal Complex Protein Zip1 Regulates the Crossover/Noncrossover Decision during Yeast Meiosis
Source: PLoS Biol. 2015 Dec 18;13(12):e1002329. doi: 10.1371/journal.pbio.1002329 (PMC4684282; doi:10.1371/journal.pbio.1002329)
Supplement: S1 Accession numbers — (DOCX) [file pbio.1002329.s001.docx]

SGD accession numbers:

*ZIP1*: S000002693

*SGS1*: S000004802

*MEK1*: S000005878

*SPO11*: S000001014

*HOP1*: S000001334

*RED1*: S000004253

*REC8*: S000006211

*MEC1*: S000000340

*TEL1*: S000000184

*RAD51*: S000000897

*DMC1*: S000000981

*ZIP2*: S000003218

*ZIP3*: S000004386

*ZIP4/SPO22*: S000001335

*MSH4*: S000001891

*MSH5*: S000002313

*SPO16*: S000001196

*TOP3*: S000004224

*RMI1*: S000005945

*MUS81*: S000002794

*MMS4*: S000000302

*YEN1*: S000000843

*CDC28*: S000000364

*CLB5*: S000006324

*CLB6*: S000003341

*CDC7*: S000002175

*DBF4*: S000002459

*CDC5*: S000004603

*NDT80*: S000001166

*MATa*: S000124955

*MATα*: S000029699

*URA3*: S000000747

*ADE2*: S000005654

*SAE2/COM1*: S000003143

*SML1*: S000004523

*ARP7*: S000006238

*RAD54*: S000003131

*ADH1*: S000005446

*MCM5*: S000004264

*MER2*: S000003782

*LYS4*: S000002642

*ARG4*: S000001060

*LEU2*: S000000523

*TRP1*: S000002414

*CTF19*: S000005939

*natMX4*:

*kanMX6*:

*hphMX4*:

Uniprot Accession numbers:

lexA: P31080

MBP: P19642

GST: P0A9D2

NCBI protein database:

*S. cerevisiae* Zip1: AAA35239.1

H. sapiens SYCP1: NP_001269470.1

M. musculus SCP-1: NP_035646.2

R. norvegicus SCP-1: Q03410.2

D. rerio SCP-1: NP_001112366.1
