# Supplementary figures and images for: Phosphorylation of the Synaptonemal Complex Protein Zip1 Regulates the Crossover/Noncrossover Decision during Yeast Meiosis
Source: PLoS Biol. 2015 Dec 18;13(12):e1002329. doi: 10.1371/journal.pbio.1002329 (PMC4684282; doi:10.1371/journal.pbio.1002329)

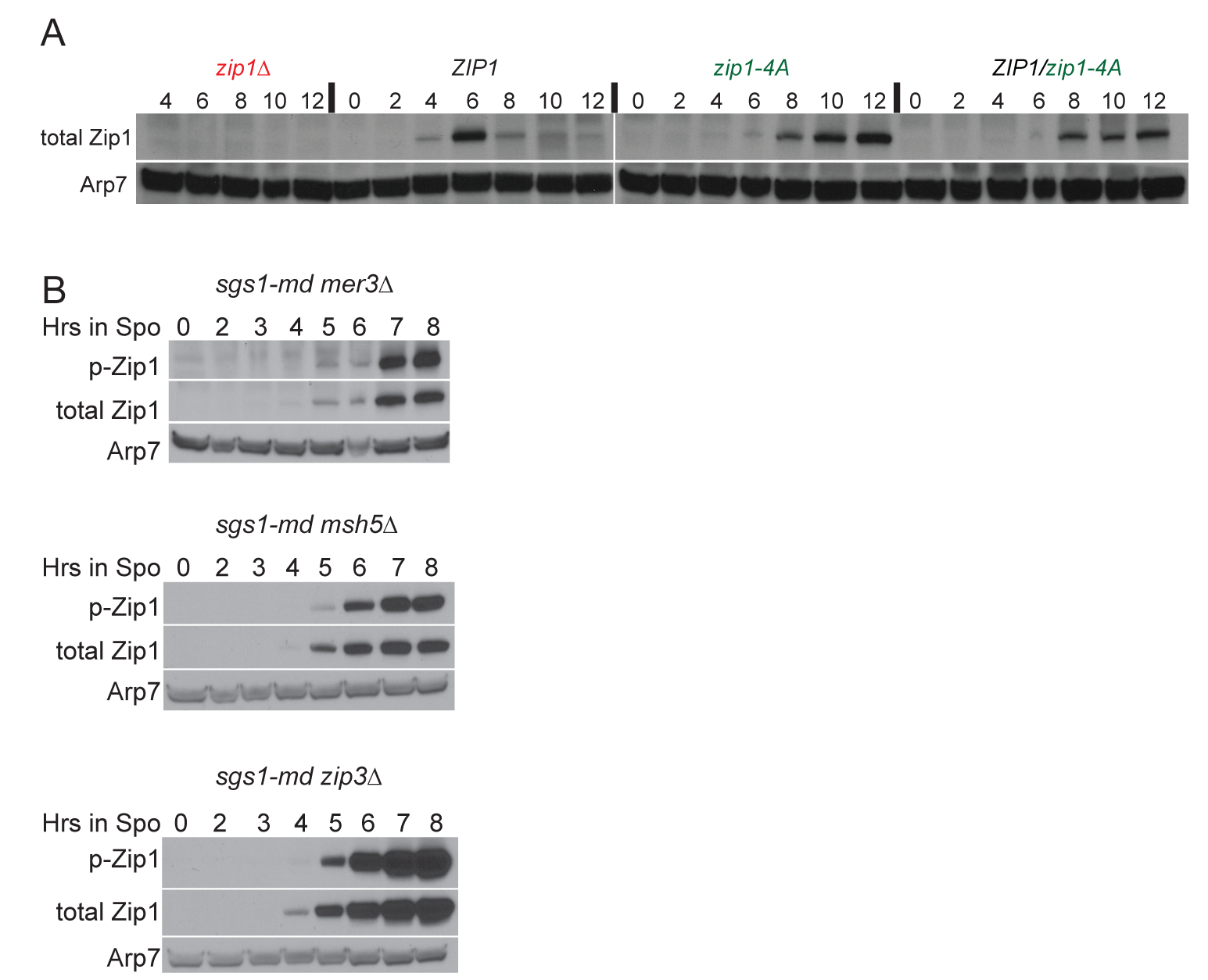

Supplement: S1 Fig — A. Time courses were performed at 30°C. Isogenic diploids containing either zip1Δ (NH2297::pRS304), ZIP1 (NH2297::p382), zip1-4A (NH2297::p382-4A), or ZIP1/zip1-4A (NH2297::p382::p382-4A) were sporulated, and protein extracts were generated for the indicated time points. Equal amounts of extract were loaded for each time point for each strain. The gels were cut in half and the top half was probed with antibodies that recognize total Zip1, while the bottom half was probed with Arp7 as a loading control. B. Zip1 S816 phosphorylation in sgs1-md diploids containing different zmm mutants. Protein extracts from time courses from the sgs1-md diploids containing either mer3Δ (NH2267::p382)), msh5Δ (NH2265::p382), or zip3Δ (NH2243::p382) were probed with either the pS816 phospho-specific antibody or the total Zip1 antibody. The Arp7 loading control was performed as described in Panel A. (TIF) [file pbio.1002329.s003.tif]

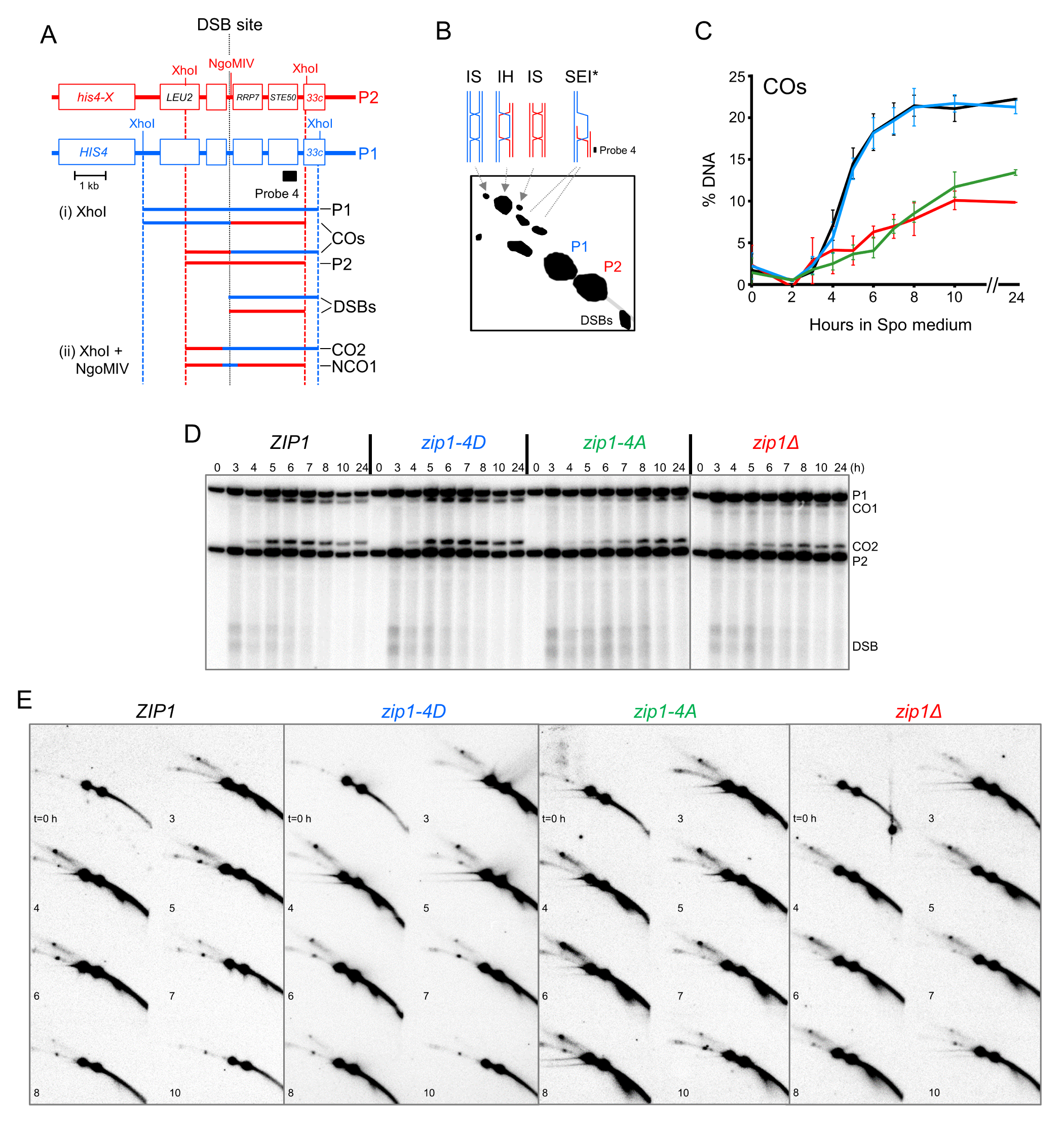

Supplement: S2 Fig — A. Upper panel: Map of restriction site polymorphisms at the HIS4::LEU2 meiotic DSB hotspot. Lower panel: Informative restriction fragments following digest with (i) XhoI or (ii) double digest with XhoI plus NgoMIV. B. Schematic representation of joint molecules detected by two-dimensional gel electrophoresis following digestion with XhoI. Asterisk: Only one of the four possible SEI species is drawn in detail (see [24]).C. Quantification of COs from two independent time courses. Error bars represent the range. D. One-dimensional Southern blot analysis of COs at the HIS4::LEU2 hotspot for the time course shown in Fig 2. DNA was digested with XhoI. E. Complete two-dimensional Southern blot analysis to monitor JMs, excerpts of which are shown in Fig 2F. (TIF) [file pbio.1002329.s004.tif]

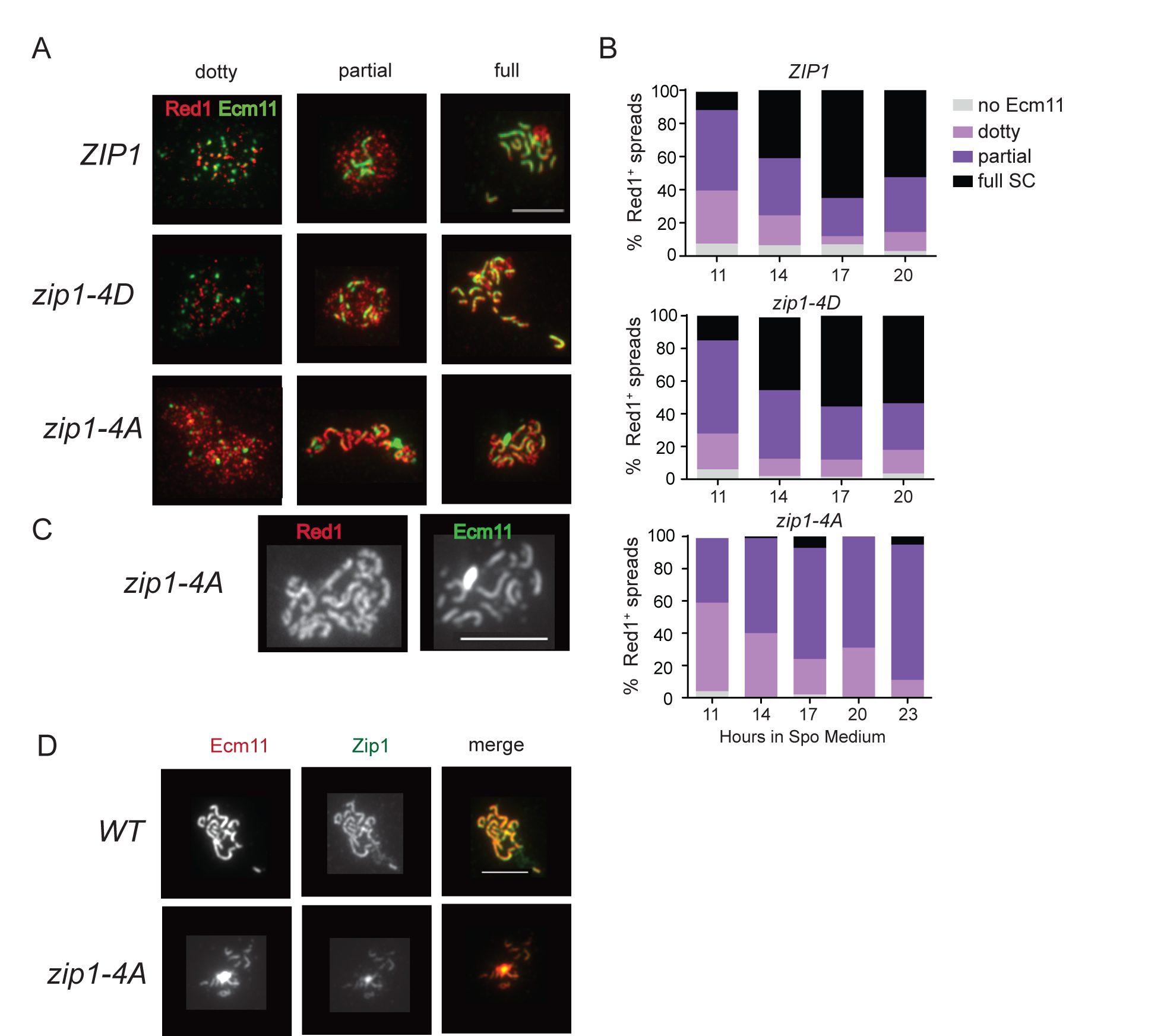

Supplement: S3 Fig — (A) Diploids from the BR background were incubated in Spo medium, and spread chromosomes were stained with anti-Red1 antibodies to visualize AEs and anti-myc antibodies to visualize the central element protein, Ecm11-myc. Red1-positive spreads were separated into categories: 1) lacking Ecm11, 2) dotty—exhibiting Ecm11 foci, 3) partial—exhibiting stretches of SC, or 4) full—exhibiting nearly complete or complete synapsis. (B) Quantification of the classes shown in Panel A. 100 spreads (zip1-4A) and 200 spreads (ZIP1, ZIP1-4D) were counted for each time point. (C) Comparison of Zip1 and Ecm11 staining in ZIP1 and zip1-4A chromosome spreads. (TIF) [file pbio.1002329.s005.tif]

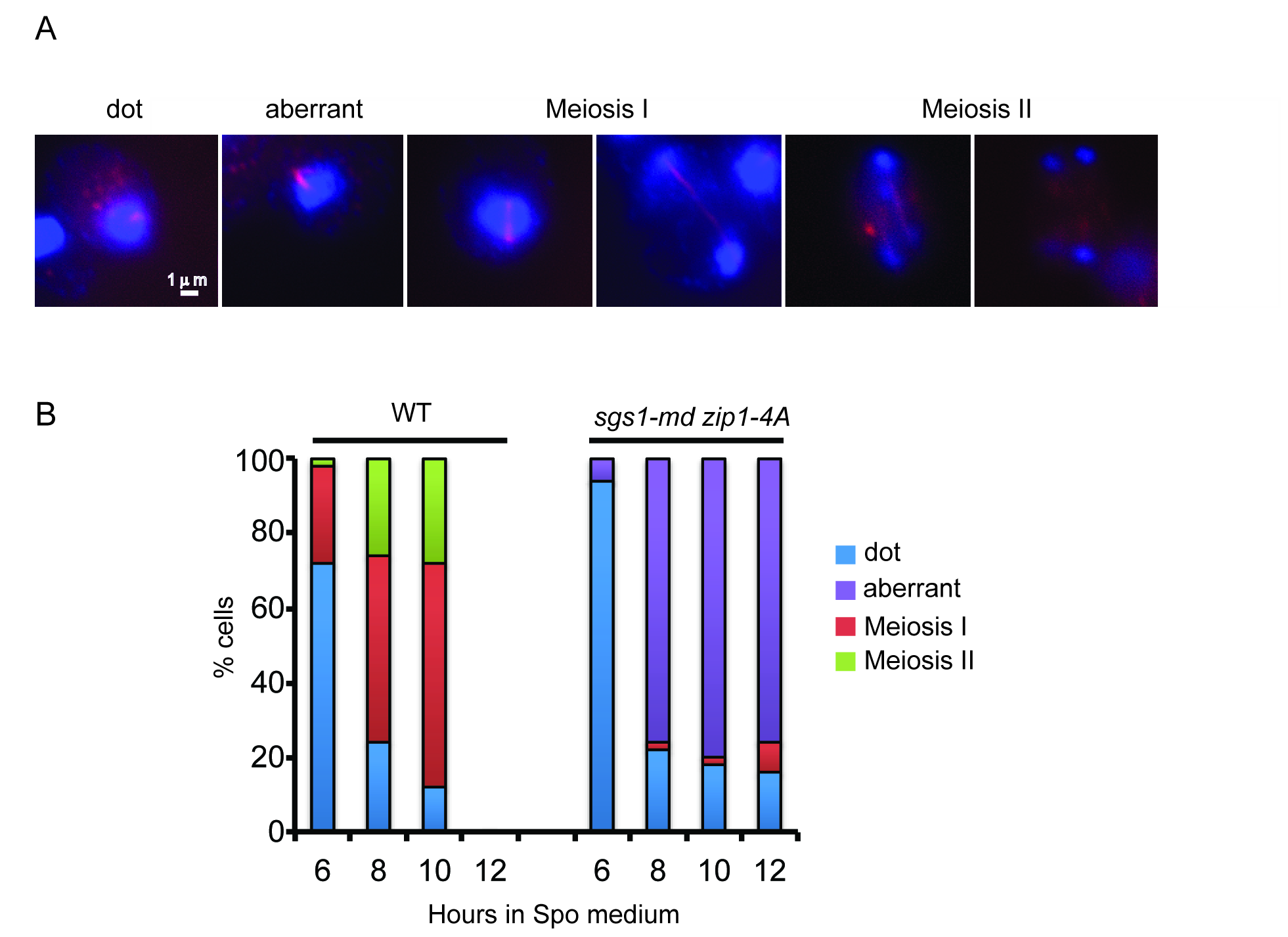

Supplement: S4 Fig — Cells from the time course in Fig 8 for WT and zip1-4A sgs1-md stained with tubulin antibodies. A. Representative pictures of cells at different stages of meiosis with the indicated spindle morphology. B. Quantitation of different spindle types in the two mutants. No tubulin staining was observed at the 0, 2, and 4 hr time points in either strain. While the WT cells progressed through both MI and MII, >90% of the zip1-4A sgs1-md cells either had very short spindles indicative of pachytene or aberrant spindles. Therefore, these cells were arrested prior to MI. (TIF) [file pbio.1002329.s006.tif]

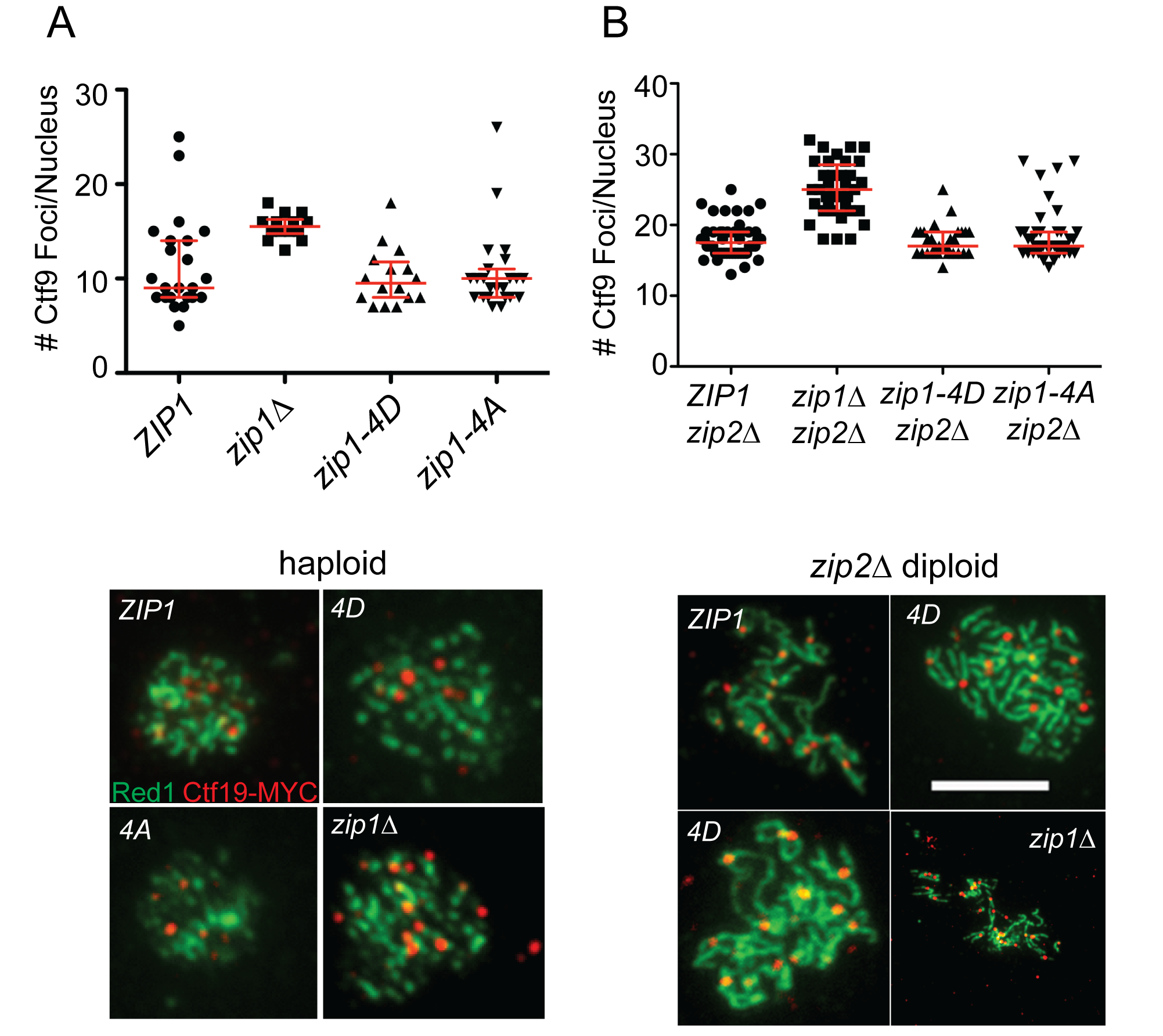

Supplement: S5 Fig — A. Nonhomologous centromere associations: The MAT a /MATα CTF19-MYC zip1Δ haploid, BR5892-8A:pB211, was transformed with either ZIP1 (p382), ZIP1-4D (p382-4D), zip1-4A (p382-4A), or pRS304 (vector). Twenty hours after transfer to Spo medium at 30°C, chromosomes were spread and stained with anti-myc (9E10) and anti-Red1 antibodies [30]. Spreads with substantial Red1 staining, indicating mid-prophase nuclei, were scored for the number of Ctf19 foci. Because haploids contain 16 nonhomologous chromosomes, pairing of nonhomologous centromeres should result in eight foci, as is observed for ZIP1 [65,66]. In contrast, zip1Δ exhibits closer to the 16 foci expected if centromeres are unpaired. Both zip1-4D and zip1-4A appear to be fully functional for nonhomologous centromere association. B. Homologous centromere associations: Chromosomes from CTF19-myc zip Δ (NH2247) carrying various ZIP1 alleles (ZIP1, zip1D, zip1-4D and zip1-4A) were spread 20 h after transfer to Spo medium and stained with anti-myc and anti-Red1 antibodies. Nuclear spreads with linear Red1 staining, indicating mid-prophase, were scored for the number of Ctf19 foci. Like zip4Δ, zip2Δ diploids exhibit ~16 foci since the chromosomes are homologously aligned and their centromeres are associated via Zip1 [66]. The zip1Δ zip2Δ mutant forms ~24 foci, consistent with the requirement for ZIP1 to hold homologous centromeres together. This number is less than the 32 foci expected if all chromosomes are unassociated, presumably due to centromere proximal axial associations. zip1-4D zip2Δ, and zip1-4A zip2Δ exhibit Ctf19 focus numbers similar to zip2Δ, indicating these alleles are functional for homologous centromere association during prophase. (TIF) [file pbio.1002329.s007.tif]
