# Supplementary material for: Phosphorylation of the Synaptonemal Complex Protein Zip1 Regulates the Crossover/Noncrossover Decision during Yeast Meiosis
Source: PLoS Biol. 2015 Dec 18;13(12):e1002329. doi: 10.1371/journal.pbio.1002329 (PMC4684282; doi:10.1371/journal.pbio.1002329)
Supplement: S1 Table — (DOCX) [file pbio.1002329.s008.docx]

**Table S1. Spore viability of various *zip1* mutants**

| Relevant  genotype | % tetrads with indicated # of viable spores | | | | | # tetrads | % spore  viability | *p* value^b^  (*ZIP1*) | *p* value  (*zip1-4A*) |
| --- | --- | --- | --- | --- | --- | --- | --- | --- | --- |
|  | 4 | 3 | 2 | 1 | 0 |  |  |  |  |
| *ZIP1*^a^ | 84.5 | 8.2 | 7.2 | 0 | 0 | 97 | 94.3 | NA^c^ | <0.0001 |
| *zip1Δ* | 24.2 | 10.2 | 25.5 | 5.1 | 35.0 | 157 | 45.9 | <0.0001 | <0.0001 |
| *zip1-4A* ^a^ | 69.0 | 10.3 | 15.5 | 4.0 | 10.9 | 174 | 76.7 | <0.0001 | NA |
| *ZIP1-4D* ^a^ | 93.3 | 1.7 | 3.4 | 0 | 0 | 119 | 96.2 | 0.249 | <0.0001 |
| *ZIP1/zip1-4A* | 87.5 | 2.9 | 9.6 | 0 | 0 | 104 | 94.5 | 0.149 | <0.0001 |
| *zip1-S815A*^a^ | 90.6 | 2.1 | 7.3 | 0 | 0 | 96 | 95.8 | 0.424 | <0.0001 |
| *zip1-S816A*^a^ | 92.3 | 3.8 | 3.8 | 0 | 0 | 104 | 97.1 | 0.074 | <0.0001 |
| *zip1-S817A*^a^ | 93.3 | 5.8 | 1.0 | 0 | 0 | 104 | 98.1 | 0.009 | <0.0001 |
| *zip1-S818A*^a^ | 86.5 | 4.8 | 8.7 | 0 | 0 | 104 | 94.5 | 0.920 | <0.0001 |
| *zip1-S815A S816A*^a^ | 66.3 | 11.5 | 11.5 | 1.0 | 9.6 | 104 | 81.0 | <0.0001 | 0.080 |
| *zip1-S815A S817A*^a^ | 68.3 | 9.6 | 14.4 | 3.8 | 3.8 | 104 | 83.7 | <0.0001 | 0.005 |
| *zip1-S815A S818A*^a^ | 79.0 | 7.0 | 10.0 | 0.0 | 4.0 | 100 | 89.2 | 0.014 | <0.0001 |
| *zip1-S816A S817A*^a^ | 75.2 | 5.9 | 11.8 | 0.7 | 6.5 | 153 | 85.6 | <0.0001 | <0.0001 |
| *zip1-S816A S818A*^a^ | 72.1 | 7.7 | 11.5 | 1.0 | 7.7 | 104 | 83.9 | <0.0001 | 0.003 |
| *zip1-S817A S818A*^a^ | 68.3 | 4.8 | 11.5 | 1.9 | 13.5 | 104 | 78.1 | <0.0001 | 0.571 |
|  |  |  |  |  |  |  |  | *p* value  (*mus8Δ ZIP1*) | *p* value  (*mus81Δ zip1-4A*) |
| *mus81Δ ZIP1*^a^ | 5.8 | 15.4 | 27.9 | 29.8 | 21.2 | 104 | 38.7 | NA | <0.0001 |
| *mus81Δ zip1Δ* ^a^ | 1.0 | 5.8 | 10.6 | 22.1 | 60.6 | 104 | 16.1 | <0.0001 | 0.116 |
| *mus81Δ zip1-4A* ^a^ | 2.0 | 8.8 | 12.7 | 22.5 | 53.9 | 102 | 20.6 | <0.0001 | NA |
| *mus81Δ ZIP1-4D* ^a^ | 7.7 | 19.2 | 24.0 | 26.9 | 22.1 | 104 | 40.9 | 0.572 | <0.0001 |

^a^ These diploids are hemizygous for the indicated *ZIP1* alleles.

^b^*p*-values were calculated using the χ^2^ calculator at <http://vassarstats.net/newcs.html>. Parentheses indicate the genotype to which the indicated strain is compared.

^c^NA = not applicable.
