# Supplementary material for: Phosphorylation of the Synaptonemal Complex Protein Zip1 Regulates the Crossover/Noncrossover Decision during Yeast Meiosis
Source: PLoS Biol. 2015 Dec 18;13(12):e1002329. doi: 10.1371/journal.pbio.1002329 (PMC4684282; doi:10.1371/journal.pbio.1002329)
Supplement: S2 Table — (DOCX) [file pbio.1002329.s009.docx]

**Table S2. *S. cerevisiae* strains**

| Strain^a^ | Genotype | Source |
| --- | --- | --- |
| NH2221 | *MAT***a** *leu2 arg4-Nsp hoΔ::LYS2 ura3 DMC1::URA3 dmc1::LEU2 mek1-as lys4Δ::hphMX4*  *MATα leu2 arg4-Nsp hoΔ::LYS2 ura3 DMC1::URA3 dmc1::LEU2 mek1-as lys4Δ::hphMX4*  *ndt80Δ::natMX4*  *ndt80Δ::natMX4* | This work |
| NH716 | *MATα leu2::hisG his4-X::LEU2(NgoMIV+ ori) hoΔ::hisG ura3(Δpst-sma)*  *MAT***a** *leu2::hisG HIS4::LEU2(BamH + oriI) hoΔ::hisG ura3(Δpst-sma)* | [1] |
| NH792 | NH716 only *dmc1Δ::kanMX6* | [2] |
| NH2188 | NH716 only *ndt80Δ::natMX4* | [2] |
| NH1054 | NH716 only *sae2Δ/com1Δ::kanMX6* | this work |
| NH1055 | NH716 only *spo11Δ::natMX4* | this work |
| NH2337 | NH716 only *spo11Δ::natMX4 ndt80Δ::hphMX4* | this work |
| NH2179^b^ | NH716 only  *MATα leu2::hisG his4-X::LEU2(NgoMIV+ ori) zip1Δ::kanMX6* *trp1-5’Δ::hphMX4*  *MATα leu2::hisG his4-X::LEU2(NgoMIV+ ori)*  *MAT***a** *leu2::hisG HIS4::LEU2(BamH + oriI)* | this work |
| NH2234^c^ | NH2179 only *ndt80Δ::natMX4* | this work |
| NH2228^b^ | NH2179 only *zip1Δ::natMX4* | this work |
| NH2230^b^ | NH2228 only *kanMX6::pCLB2-3HA-SGS1* | this work |
| NH2242^b^ | NH2230 only *zip2Δ::KlURA3* | this work |
| NH2243^b^ | NH2230 only *zip3Δ::KlURA3* | this work |
| NH2249^b^ | NH2230 only *spo11Δ::KlURA3* | this work |
| NH2265^b^ | NH2230 only *msh5Δ::KlURA3* | this work |
| NH2266^b^ | NH2230 only *sae2Δ::KlURA3* | this work |
| NH2267^b^ | NH2230 only *mer3Δ::KlURA3* | this work |
| NH2271^b^ | NH2230 only *spo16Δ::KlURA3* | this work |
| NH2305^b^ | NH2230 only *zip4Δ::KlURA3* | this work |
| NH2297 ^c^ | same as NH716 only *zip1Δ::kanMX6 trp1-5’Δ::hphMX4* | this work |
| NH2298^c^ | same as NH716 only *zip1Δ::natMX4* *trp1-5’Δ::hphMX4* | this work |
| NH2299^c^ | same as NH716 only *zip1Δ::natMX4* *trp1-5’Δ::hphMX4 kanMX6::pCLB2-3HA-SGS1* | this work |
| NH2300^b^ | same as NH716 only *zip1Δ::kanMX6* *trp1-5’Δ::hphMX4 mus81Δ::natMX4* | this work |
| NH2322 | same as NH716 only *cdc7-as3-9myc* | this work |
| NH2309-6-3 | *MATα leu2 arg4-Nsp lys2 hoΔ::LYS2 cdc7-as3-9myc mcm5-bob1 ura3 trp1-5’Δ::hphMX4* | this work |
| NHY957 | *MAT***a** *CENIII::ADE2 leu2::hisG his4-B* *trp5-S CYH2 met13-B LYS5* *can1 ura3(ΔSma-Pst)*  *MATα CENIII LEU2 HIS4* *TRP1 cyh2 MET13 lys5-P* *CAN1 ura3(ΔSma-Pst)*  *CENVIII::URA3 thr1-a cup1* *ade2* *ho::hisG TRP1*  *CENVIII THR1 CUP1 ade2 ho::hisG TRP1* | [3] |
| NH2241^b^ | *MAT***a** *CENIII::ADE2 leu2::hisG his4-B* *trp5-S CYH2 met13-B LYS5* *can1 ura3(ΔSma-Pst)*  *MATα CENIII LEU2 HIS4* *TRP1 cyh2 MET13 lys5-P* *CAN1 ura3(ΔSma-Pst)*  *CENVIII::URA3 thr1-a cup1* *ade2* *ho::hisG TRP1* *zip1Δ::natMX4*  *CENVIII THR1 CUP1 ade2 ho::hisB trp1-5’Δ::hphMX4::TRP1 zip1Δ::natMX4* | this work |
| NH144 | *MAT***a** *leu2ΔhisG his4-x ARG4 ura3 lys2 hoΔ::LYS2*  *MATα leu2-K HIS4 arg4-Nsp ura3 lys2 hoΔ::LYS2* | [4] |
| NH774^d^ | NH144 only *sml1Δ::HA mec1(∆1632-2368)::kanMX6* | this work |
| NH742^d^ | NH144 only *tel1(Δ2381-2787)::kanMX6* | this work |
| NH2072 | NH144 only *CDC7-3FLAG::kanMX6 ndt80Δ::hphMX4* | this work |
| NH2082 | NH144 only *cdc7-as-3FLAG::kanMX6 ndt80Δ::hphMX4* | this work |
| NH2309-6-3 | *MATα leu2 arg4-Nsp lys2 hoΔ::LYS2 cdc7-myc-as3 mcm5-bob1 ura3 trp1-5’Δ::hphMX4* | this work |
| NH2311 | *MAT***a** *leu2::hisG ho::LYS2 lys2 ura3 his3::hisG trp1::hisG::ZIP1::TRP1 zip1::LEU2*  *MATα leu2::hisG ho::LYS2 lys2 ura3 his3::hisG trp1::hisG::ZIP1::TRP1 zip1::LEU2*  *SPO11-PROTEIN A::S.pombe his5*^+^  *SPO11-PROTEIN A::S.pombe his5^+^* | this work |
| NH2312 | NH2311 only *TRP1 zip1::LEU2* | this work |
| NH2313 | NH2311 only *zip1-4A* | this work |
| NH2314 | NH2311 only *ZIP1-4D* | this work |
| NH520/pLW3 | NH144 only *dmc1Δ::LEU2* *mek1::kanX6/2μ URA3 GST-mek1-as* | [5] |
| NH566::pLT11::  pXC6 | *MAT***a** *leu2 HIS4 hoΔ::LYS2 arg4-Nsp hop1::LEU2 mek1Δ::LEU2 ura3::HOP1::URA3*  *MATα leu2 his4 hoΔ::LYS2 arg4-Nsp hop1::LEU2 mek1Δ::LEU2 ura3*  *ade2::mek1-K199R-ADE2*  *ade2* | this work |
| BR5892-8A::pB211*^b^ | *MATα leu2-3,112 trp1-289 ura3-1 thr1-4::MATa THR1 ade2-1 CTF10-myc-kanMX6 zip1Δ::URA3 spo1∆::ADE2* | this work |
| NH2247*^b^ | *MAT***a** *leu2-3,112 his4-260 trp1-289 thr1-4 arg4 lys2 ade2-1 ura3 CTF19-myc-kanMX6*  *MATα leu2-3,112 his4-260 trp1-289 thr1-4 ARG4 lys2 ade2-1 ura3 CTF19-myc-kanMX6*  *zip1Δ::URA3 zip2Δ::LEU2*  *zip1Δ::URA3 zip2Δ::LEU2* | this work |
| BR5894 ECM11-myc-kan*^b^ | *MAT***a** *leu2-3,112 his4-260 trp1-289 thr1-4 ade2-1 ECM11 zip1Δ::URA3*  *MATα leu2-3,112 his4-260 trp1-289 thr1-4 ade2-1 ECM11-myc-kan zip1Δ::URA3* | this work |

^a^All strains are derived from the SK1 strain background, except those marked with an asterisk, which are derived from the BR strain background.

^b^Strains contain a single copy of either pRS304, p382 (*ZIP1*), p382-4A (*zip1-4A*) or p382-4D (*zip1-4D*) integrated at *trp1*.

^c^ Strains are homozygous for either pRS304, p382 (*ZIP1*), p382-4A (*zip1-4A*) or p382-4D (*zip1-4D*) integrated at *trp1.*

^d^The *MEC1* deletion removes the PI3/PI4 kinase domain located between 2074-2316. The *TEL1* deletion removes the PI3/PI4 kinase domain between 2459-2704.

1. Callender TL, Hollingsworth NM (2010) Mek1 suppression of meiotic double-strand break repair is specific to sister chromatids, chromosome autonomous and independent of Rec8 cohesin complexes. Genetics 185: 771-782.

2. Liu Y, Gaines W, Callender TL, Oke A, Busygina V, et al. (2014) Down-regulation of Rad51 activity during meiosis in yeast prevents competition with Dmc1 for repair of double-strand breaks. PLoS Genetics 10: e1004005.

3. de los Santos T, Hunter N, Lee C, Larkin B, Loidl J, et al. (2003) The Mus81/Mms4 endonuclease acts independently of double-Holliday junction resolution to promote a distinct subset of crossovers during meiosis in budding yeast. Genetics 164: 81-94.

4. Hollingsworth NM, Ponte L, Halsey C (1995) *MSH5*, a novel MutS homolog, facilitates meiotic reciprocal recombination between homologs in *Saccharomyces cerevisiae* but not mismatch repair. Genes Dev 9: 1728-1739.

5. Wan L, de los Santos T, Zhang C, Shokat K, Hollingsworth NM (2004) Mek1 kinase activity functions downstream of *RED1* in the regulation of meiotic DSB repair in budding yeast. Mol Biol Cell 15: 11-23.
